# Developmental and Cellular Basis of Vertical Bar Color Patterns in the East African Cichlid Fish *Haplochromis latifasciatus*

**DOI:** 10.3389/fcell.2020.00062

**Published:** 2020-02-11

**Authors:** Yipeng Liang, Jan Gerwin, Axel Meyer, Claudius F. Kratochwil

**Affiliations:** Zoology and Evolutionary Biology, Department of Biology, University of Konstanz, Konstanz, Germany

**Keywords:** vertical bars, pigment development, gene expression, chromatophores, Cichlidae

## Abstract

The East African adaptive radiations of cichlid fishes are renowned for their diversity in coloration. Yet, the developmental basis of pigment pattern formation remains largely unknown. One of the most common melanic patterns in cichlid fishes are vertical bar patterns. Here we describe the ontogeny of this conspicuous pattern in the Lake Kyoga species *Haplochromis latifasciatus*. Beginning with the larval stages we tracked the formation of this stereotypic color pattern and discovered that its macroscopic appearance is largely explained by an increase in melanophore density and accumulation of melanin during the first 3 weeks post-fertilization. The embryonal analysis is complemented with cytological quantifications of pigment cells in adult scales and the dermis beneath the scales. In adults, melanic bars are characterized by a two to threefold higher density of melanophores than in the intervening yellow interbars. We found no strong support for differences in other pigment cell types such as xanthophores. Quantitative PCRs for twelve known pigmentation genes showed that expression of melanin synthesis genes *tyr* and *tyrp1a* is increased five to sixfold in melanic bars, while xanthophore and iridophore marker genes are not differentially expressed. In summary, we provide novel insights on how vertical bars, one of the most widespread vertebrate color patterns, are formed through dynamic control of melanophore density, melanin synthesis and melanosome dispersal.

## Introduction

Pigment patterns play important roles in many aspects of animal biology. Yet, until now, only in a few “model” organisms we do have insights into the molecular and developmental underpinnings of color pattern formation and evolutionary diversification. Among teleosts, the zebrafish *Danio rerio* and the Medaka *Oryzias latipes* are the main model organisms for investigation of pigmentation (Meyer et al., 1993, 1995; Kelsh et al., 1996; Nagao et al., 2014; Irion and Nüsslein-Volhard, 2019; Patterson and Parichy, 2019). More recently, African cichlid fishes with their richness in color patterns are increasingly studied to understand the molecular mechanisms of color pattern formation including but not limited to egg spot patterns (Henning and Meyer, 2012; Santos et al., 2014, 2016), blotch patterns (Streelman et al., 2003; Roberts et al., 2009), amelanism (Kratochwil et al., 2019b), horizontal stripe patterns (Ahi and Sefc, 2017; Kratochwil et al., 2018; Hendrick et al., 2019) and pigment distribution more generally (Albertson et al., 2014). And although progress has been made identifying target genes and loci that drive evolutionary diversification in cichlids (Roberts et al., 2009; Kratochwil et al., 2018) and play key roles in adaptation and speciation (Seehausen et al., 1999; Elmer et al., 2009; Maan and Sefc, 2013), the developmental and cellular mechanisms of pigmentation phenotypes have been barely studied.

Pigment patterns are ultimately caused by spatial variation in pigmentary and/or structural tissue properties. Those can be generated by different distribution, density and aggregation state of pigment cells (chromatophores) and their multi-layered arrangement, as well as variation in the synthesis and arrangement of light-absorbing pigments or molecules causing structural coloration (Irion and Nüsslein-Volhard, 2019; Patterson and Parichy, 2019). In teleosts several types of chromatophores, including melanophores, xanthophores, iridophores, erythrophores, leucophores, and cyanophores have been described (Burton and Burton, 2017). Melanophores (containing the brown to black pigment melanin), xanthophores/erythrophores (containing yellow to red pigments) and iridophores (containing reflective guanine platelets causing structural coloration) have been also found in cichlids (Maan and Sefc, 2013).

For the mechanisms of color pattern formation, mainly (horizontal) stripe patterns have received attention because the most commonly studied “model” teleost, the zebrafish, carries this characteristic pattern. Vertical bars are less studied, with the exception of recent detailed description of the convict cichlid *Amatitlania nigrofasciata* (Prazdnikov and Shkil, 2019), studies in Amphiprioninae, the anemone fishes (Salis et al., 2018; Roux et al., 2019) and Lake Malawi cichlids (Hendrick et al., 2019). Bar patterns are presumably adaptive as such disruptive coloration breaks the outline of the individual and thereby constitutes a form of camouflage, in particular in visually complex habitats (Seehausen et al., 1999; Maan and Sefc, 2013). Additionally color patterns are often involved in species recognition (Hemingson et al., 2019).

**FIGURE 1:**
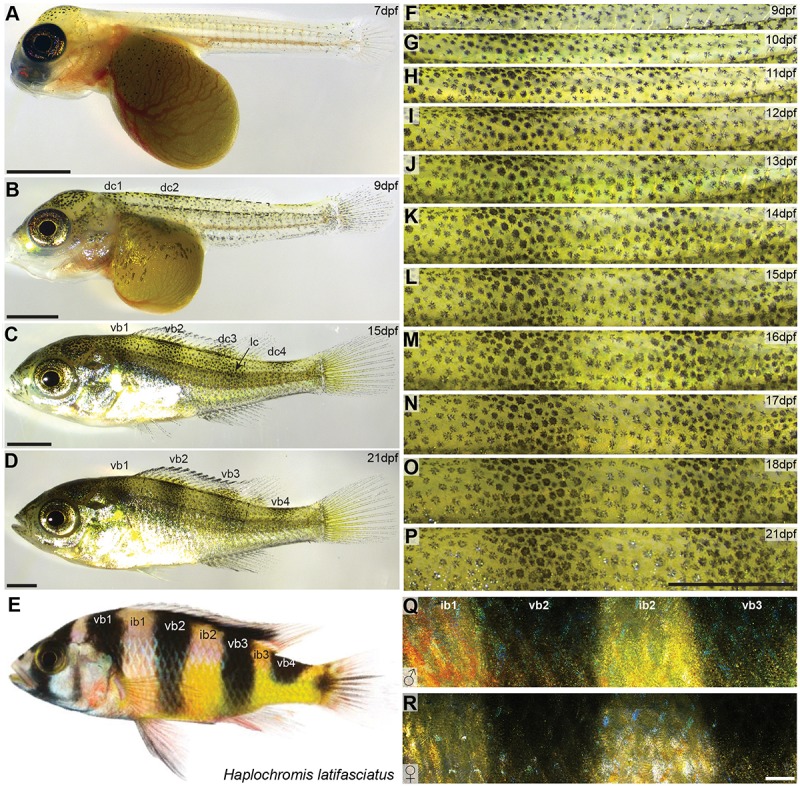
Vertical bar patterns in *Haplochromis latifasciatus*. **(A–E)** Developmental stages of *H. latifasciatus* at 7 dpf **(A)**, 9 dpf **(B)**, 15 dpf **(C)**, 21 dpf **(D)** and an adult individual **(E)**. At 7 dpf Melanophores are mainly located on the head and yolk sac **(A)**. At 9 dpf, two melanophore dorsal clusters (dc1 and dc2) are appearing indicating the position of vb1 and vb2, respectively **(B)**. The adult vertical pattern is already fully formed at 15dpf **(C)** and further increases in contrast until 21dpf **(D)** and beyond. Adult *H. latifasciatus* are characterized by four dark vertical bars **(E)**. The region between the bars (interbars) have yellow to orange-red hues. **(F–R)** High magnification microscopy images showing the development of ib1, vb2, ib2, and vb3 from 9 dpf to 21 dpf **(F–P)** and adult male **(Q)** and female **(R)**. Interbar melanophores are often lighter, with parts of the chromatophore or the center being almost pigment-free **(F–P)**. Vertical bar pattern does not differ between sexes in adults **(Q,R)**. Abbreviations: dc: dorsal cluster, lc: lateral cluster, vb: vertical bar, ib: interbar. Scale bars are 1 mm in **(A–D,F–P)**, 2 mm in **(Q,R)**.

Here, we focus on the vertical bar pattern of *Haplochromis latifasciatus* (Figure 1E) from Lake Kyoga, a lake north of Lake Victoria. *H. latifasciatus* is a species of the haplochromine cichlids, the most species-rich cichlid lineage that forms the adaptive radiations of Lake Victoria and Malawi with 500 and 800 species respectively. We describe the formation of the pattern during development and compare it to other teleosts, characterize which cell types and properties underlie this conspicuous pattern, and use a candidate gene approach to obtain insights into the underlying molecular mechanisms.

## Results

### Developmental Progression of Vertical Bar Formation in *H. latifasciatus*

Both male and female *H. latifasciatus* in adult stage are characterized by four (in some strains five) vertical melanic bars. Individuals of our breeding stock had consistently four bars (*n* > 30; Figure 1E): one anterior bar above the operculum (vertical bar 1; vb1), two vertical bars in the trunk area that cover the whole dorso-ventral axis (vb2 and vb3) and a more posterior vertical bar (vb4) at the anterior caudal peduncle that only covers the dorsal part up to the horizontal myoseptum. The regions between the bars (referred to as interbars, ib) are yellow to beige with dominant males often having nuptial colors with stronger yellow but anteriorly also red to orange hues (Figures 1Q,R). In contrast to the closely related Lake Victoria cichlids bars are thicker and more pronounced in *H. latifasciatus* with a clearer demarcation, lower number and without as pronounced physiological color change (Greenwood, 1974; Seehausen et al., 1999).

To investigate the formation of vertical bars (Figure 1E), we described the development of *H. latifasciatus* larvae between 7 and 21 days post-fertilization (dpf; Figures 1A–D,F–P and Supplementary Figure S1). The developmental progression of vertical bar formation is fully consistent in all three individuals examined (Supplementary Figures S1, S2). At 7 dpf, melanophores are present in the dorsal head region as well as on the dorsal part of the yolk sac. In the trunk area only a few melanophores have formed without any obvious indication of a bar-like pattern (Figure 1A). Starting at 8 dpf vertical bars form in an anterio-posterior sequence (Figures 1B–D,F–P and Supplementary Figure S1). Trunk melanophore number has increased considerably within an anterior dorsal patch (dc1) forming at 8 dpf (data not shown), followed by a more posterior one at 9 dpf (dc2; Figure 1B). The melanophore patches anticipate the position of vb1 and vb2, respectively (Figures 1B,C). At 10 dpf a third and fourth dorsal patch (dc3 and dc4) are appearing at the positions where vb3 and vb4 will later form, respectively (Figures 1C,G and Supplementary Figures S1, S2). After the appearance of the dorsal patches they expand dorsally into the dorsal fin and ventrally forming the four bars. One exception is the posterior bar vb4, where the bar only extends to the horizontal myoseptum. The formation of vb3 is furthermore contributed by a second melanophore cluster (lateral cluster; lc) that forms in a more posterior-ventral position and merges with the developing bar between 12 and 13 dpf (Figures 1C,I,J and Supplementary Figures S1, S2). After this time, at around 2 weeks post-fertilization, the complete adult vertical pattern is already fully formed, but the contrast of the bars further increases until 21 dpf (Figures 1D,P and Supplementary Figure S1) and beyond.

### Cellular Correlates of Vertical Bar Formation

In order to understand the formation of the characteristic bar pattern of *H. latifasciatus*, we analyzed the progression of the bar pattern formation over time. Specifically, we analyzed how the darkening of the bar regions is generated at the cellular level. We hypothesized that three processes might contribute to the darker appearance of the bar regions: (a) melanosome dispersal (the aggregation and dispersion of melanosomes, the melanin-containing organelles, within melanophores) as it increases the fraction of the tissue covered by melanin, (b) the density of melanophores, and (c) the darkness of the melanophores, i.e., the concentration of melanin.

**FIGURE 2:**
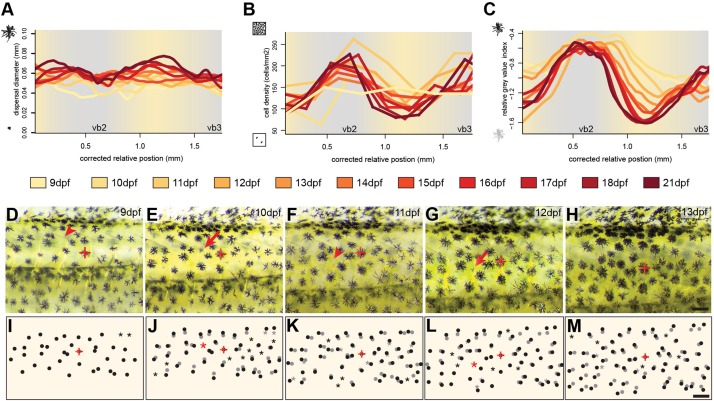
Cellular changes during vertical bar formation. **(A–C)** Measurement of melanosome dispersal diameter, melanophore density and the darkness of the melanophores focusing on the dorsal portion of vb2, ib2, and vb3 illustrate changes in chromatophore number and characteristics during bar pattern formation between 9 and 21 dpf. No obvious difference could be found in melanophore dispersal diameter between bars and interbars **(A)**. The melanophore density increases in bars compared to interbars **(B)**. Relative gray values of melanophores increase as well **(C)**. **(D–M)** Time-lapse vertical bar development in the same individual from 9 to 13 dpf as photographs **(D–H)** and schematics **(I–M)**. The melanophore with the red cross in **(D–M)** was used to align the images. Dots show the position of melanophores in **(I–M)**. Position on the previous days (based on image overlay) are labeled in gray. Red arrowhead in **(D–H)** and asterisks in **(I–M)** indicate where the formation of a new melanophore will occur, red arrows in **(D–H)** and black dots next to a gray asterisk in **(I–M)** the forming melanophore on the consecutive day. Scale bars are 0.5 mm in **(D–M)**.

To investigate the development and the importance of these factors we followed the development of three individuals over twelve days of development between 9 and 21 dpf focusing on the dorsal portion of the two bars in the trunk region, vb2 and vb3 and the yellow interbar region in between (ib2). To assess melanosome dispersal, we calculated the diameter of the melanin-covered part of all melanophores (see section Materials and Methods). We found no strong spatial difference in melanosome aggregation (Figure 2A and Supplementary Figure S1), yet dispersal diameters increased with age, likely because cells are still growing during these early developmental stages (Supplementary Figure S3). Cell density was evenly distributed at 9 dpf, but during the formation of the bars, cells became more densely packed in the bar regions, while cell density decreased in the interbar regions (Figure 2B and Supplementary Figure S1). To measure the darkness of individual melanophores we measured relative gray values (see section Materials and Methods). Here the difference between bar and interbar regions continuously increased suggesting stronger accumulation of melanin in bar melanophores (Figure 2C and Supplementary Figure S1). Indeed, closer observation of melanophores shows that interbar melanophores are often not fully filled with melanin, with parts of the chromatophore, often the center of the cell being poorly pigmented (Figures 1N–P and Supplementary Figure S1).

Next, we investigated the cellular behavior of single melanophores between 9 and 13 dpf. To do so, we photographed the vb2 region of the same individual on five consecutive days and tracked cellular migration and the formation of new melanophores (Figures 2D–M). The data suggests that melanophores mainly move indirectly through the general expansion of the skin. Newly differentiating melanophores could be found across the whole examined area, but at an increased rate in the forming bars. They grow to the full size (diameter: ∼0.06 mm) within 1–2 days. We found no evidence of proliferating melanin-containing cells.

In summary, these results suggest that the bars of *H. latifasciatus* form through spatial variation in melanophore properties (i.e., melanin content) and melanophore cell density that mainly arise in the second week after hatching (standard length 6–8 mm). The increase in melanophore density is most likely caused by an increased differentiation of melanoblasts within the bar regions.

### Adult Patterns in *H. latifasciatus*

**FIGURE 3:**
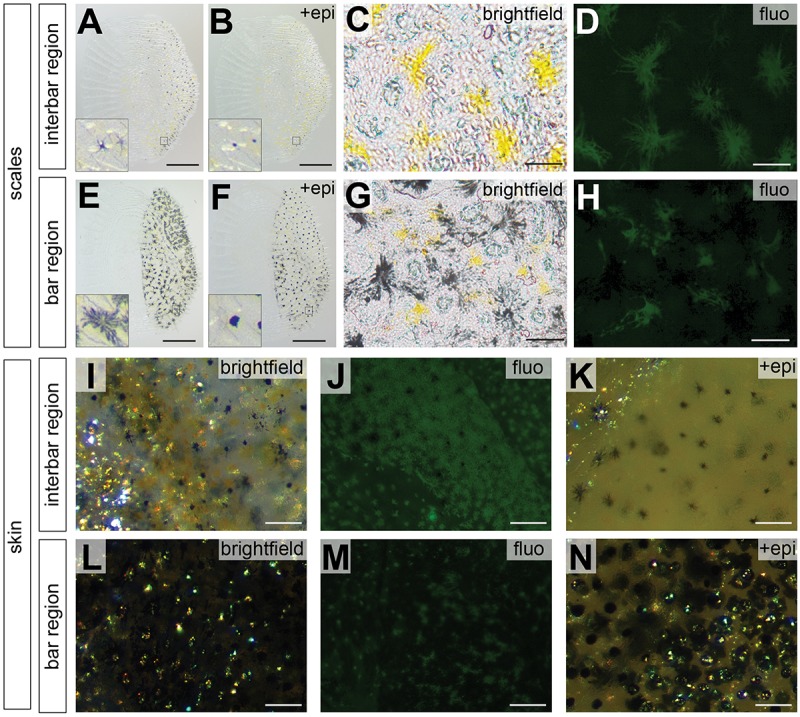
Photographs of scale and skin dissections in bar and interbar region. **(A–H)** Photographs of scales from interbar region **(A–D)** and bar region **(E–H)**. Melanophores from scales of interbars **(A)** and bars **(E)** aggregate after epinephrine treatment **(B,F)** allowing accurate quantification. Insets of **(A,B,E,F)** show the single melanophores before and after epinephrine treatment. Xanthophores (yellow) from scales of interbars **(C)** and bars **(G)** were detected via their autofluorescence **(D,H)**. **(I–N)** High magnification photographs skin, where scales have been removed. Brightfield images of interbar **(I)** and bar **(L)** fluorescent images of interbar **(J)** and bar **(M)**; brightfield images after epinephrine treatment for interbar **(K)** and bar region **(N)**. Scale bars are 500 μm in **(A,B,E,F)**; 50 μm in **(C,D,G,H)**; 100 μm in **(I–N)**.

To investigate the distribution of chromatophores in different integument regions, we estimated cell density and size of both, melanophores and xanthophores in the two interbars (ib1, ib2) and the two bars (vb2, vb3) of the trunk region (Figure 1E). Here, we only quantified pigment cells in female individuals as the vertical bar pattern does not differ between sexes, but substantial interindividual variation in red coloration of males would have complicated quantifications (Figures 1Q,R). Scales and dermis without scales (from hereon called “scales” and “skin”, respectively) were analyzed separately. Three types of chromatophores could be found in both the dark bars and the light interbars: melanophores with black/dark brown pigments, xanthophores with yellow to orange and reddish pigments (we did not differentiate between xanthophores and erythrophores; see discussion in section Materials and Methods), and iridophores with iridescent/reflective properties (Figures 3I,L). To quantify pigment cell quantity and properties we used three measurements: (a) pigment cell coverage, (b) pigment cell dispersion, and (c) pigment cell density.

**FIGURE 4:**
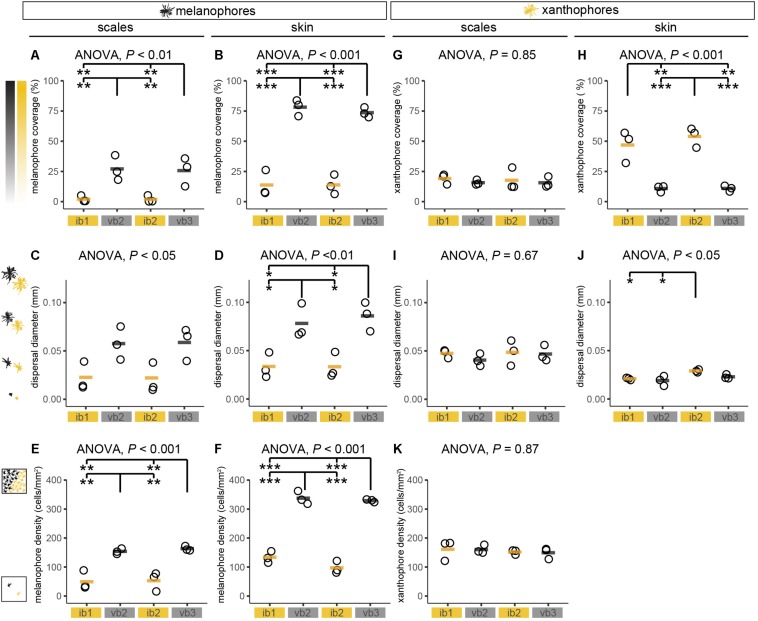
Chromatophore measurements in adult *H. latifasciatus*. **(A–K)** Chromatophore coverage **(A,B,G,H)**, dispersal diameter of cells **(C,D,I,J)** and cell density **(E,F,K)** of melanophores **(A–F)** and xanthophores **(G–K)** were estimated in scales and skin from two interbars (ib1 and ib2) and two bars (vb2 and vb3). *P*-values are based on ANOVA and Tukey–Kramer *post hoc* tests. Each dot represents the mean value of one individual (full data see Supplementary Figures S4–S6). Black/orange lines depict the mean of the three individuals. ***P < 0.001; **P < 0.01; *P < 0.05.

Pigment cell coverage is influenced by both cell number and size (or intracellular dispersal of pigments in the cell) and was measured by estimating the percentual coverage of the tissue with pigment using light microscopy for melanophores and fluorescence microscopy to detect the autofluorescence of xanthophores. Consistent with the visual impression, melanophore coverage in bars was significantly higher than in the yellowish interbars in both scales (26.4% in bars, 2.0% in interbars) and skin (75.9% in bars, 13.7% in interbars) (Figures 3, 4A,B, Supplementary Figure S4, and Supplementary Table S1). Both melanophore density and melanosome dispersal contributed to the difference in melanophore coverage. The average melanosome dispersal diameter is larger in dark bars (Ø 0.058 mm in scale, Ø 0.082 mm in skin) than light interbars (Ø 0.022 mm in scale, Ø 0.033 mm in skin) (Figures 4C,D, Supplementary Figure S5, and Supplementary Table S1). However, variation within the same skin region was quite high suggesting that both dispersed and aggregated melanophores are widely distributed (Supplementary Figure S5 and Supplementary Table S1). The density of melanophores in both scale and skin were significantly higher in the bars (158 cells/mm^2^ in scale, 333 cells/mm^2^ in skin) than in interbars (52 cells/mm^2^ in scale, 115 cells/mm^2^ in skin) (Figures 4E,F, Supplementary Figure S6, and Supplementary Table S1).

Xanthophore coverage in bars and interbars is similar on scales (15.7% in bars, 18.4% in interbars), but significantly different in the skin (10.9% in bars, 50.4% in interbars) (Figures 3D,H,J,M, 4G,H, Supplementary Figure S4, and Supplementary Table S1). However, xanthophore coverage might be underestimated in the skin of the bar regions due to the high density of melanophores that could aggravate detection of the xanthophore autofluorescence. The dispersal diameter of xanthophores did not differ between interbars (Ø 0.048 mm in scale, Ø 0.025 mm in skin) and bars (Ø 0.044 mm in scale, Ø 0.021 mm in skin), neither in scales nor in skin preparations (Figures 4I,J, Supplementary Figure S5, and Supplementary Table S1). Cell densities were comparable in scales of interbars (157 cell/mm^2^) and bars (155 cell/mm^2^) (Figure 4K, Supplementary Figure S6, and Supplementary Table S1).

### Gene Expression Associated With the Bar Patterns in *H. latifasciatus*

**FIGURE 5:**
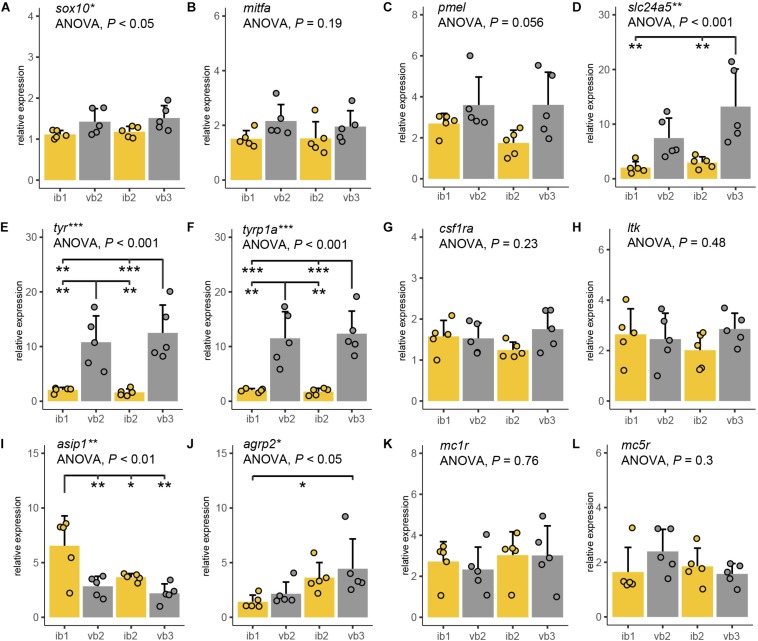
Expression differences of pigmentation candidate genes. **(A–K)** Quantitative PCR of *sox10* **(A)**, *mitfa* **(B)**, *pmel* **(C)**, *slc24a5* **(D)**, *tyr* **(E)**, *tyrp1a* **(F)**, *csf1ra* **(G)**, *ltk* **(H)**, *asip1* **(I)**, *agrp2* **(J)**, *mc1r* **(K),** and *mc5r* **(L)** mRNA levels along the anterior-posterior axis of adult *H. latifasciatus* including two vertical bars (gray bar plots, vb2, vb3) and the light interbar regions (yellow bar plots; ib1 and ib2). Differences were tested by ANOVA followed by Tukey–Kramer *post hoc* test, *n* = 5 (individual dots). Error bars indicate means + SD. Abbreviations: ***P < 0.001; **P < 0.01; *P < 0.05.

Next, we analyzed the molecular correlates of the observed differences in pigment cell density and pigment synthesis. Molecular markers for iridophore and xanthophore were also used as we could not analyze differences in these cells’ number due to the high melanophore density in the bar regions. Therefore, we compared expression levels of twelve candidate genes across the same two bar and interbar regions for the chromatophore measurement (ib1, vb2, ib2, vb3) using quantitative real-time PCR (qPCR; Figure 5 and Table 1). The selected genes are including marker genes for chromatophores, melanin synthesis genes and genes involved in the melanocortin signaling pathway. The latter was a particular focus as they have been previously implicated in color pattern formation of cichlids, teleosts and vertebrates more generally (Table 1).

**TABLE 1:** Selected candidate genes involved in coloration and pigment patterns in fish.

**Gene**	**Full name and described function**	**References**
*agrp2*	*agouti related peptide 2*: A member of the agouti gene family that has been shown to repress (horizontal) stripe formation in cichlids. It is also involved in background matching in zebrafish.	Zhang et al., 2010; Shainer et al., 2017; Kratochwil et al., 2018
*asip1*	*agouti signal peptide 1*: Another member of the agouti gene family that regulates dorsal-ventral pigmentation in the fish skin.	Cerda-Reverter et al., 2005; Kurokawa et al., 2006; Guillot et al., 2012; Cal et al., 2017, 2019
*csf1ra*	*colony stimulating factor 1 receptor a*: A *kit*-related tyrosine receptor kinase, which is essential for migration and survival of xanthophores.	Parichy et al., 2000; Parichy and Turner, 2003
*ltk*	*leukocyte receptor tyrosine kinase*: A tyrosine kinase receptor that is crucial for fate specification of iridophores from neural crest cells.	Lopes et al., 2008
*mc1r*	*melanocortin 1 receptor*: A G-protein-coupled seven transmembrane helix receptor that regulate several pigment cell specific processes. Loss-of-function mutation are associated with loss of melanic pigmentation.	Selz et al., 2007; Richardson et al., 2008
*mc5r*	*melanocortin 5 receptor*: Another melanocortin receptors expressed in both melanophore and xanthophores.	Kobayashi et al., 2010, 2012a
*mitfa*	*microphthalmia-associated transcription factor a*: A master regulator of melanophore/melanocyte differentiation across vertebrates.	Lister et al., 1999; Béjar et al., 2003
*pmel*	*premelanosome protein a*: A melanosome protein that plays an important role in the structural organization of premelanosomes and the formation of intraluminal fibrils during melanosome biogenesis.	Chakraborty et al., 1996; Béjar et al., 2003; Du et al., 2003; Schonthaler et al., 2005; Theos et al., 2005
*slc24a5*	*solute carrier family 24 member 5*: A transporter protein localized in the melanosomal membrane that is essential for melanin synthesis.	Lamason et al., 2005
*sox10*	*sex determining region Y box 10*: A transcription factor that is essential for the specification of chromatophore progenitors.	Dutton et al., 2001; Elworthy et al., 2003; Hou et al., 2006
*tyr*	*tyrosinase*: An oxidase that can controls the melanogenesis as the rate-limiting enzyme by catalyzing tyrosine into dopaquinone via L-dopa.	Korner and Pawelek, 1982; Hidehito et al., 1994; Camp and Lardelli, 2001
*tyrp1a*	*tyrosinase-related protein 1a*: Another enzyme of the tyrosinase family that catalyzes the melanin biosynthesis.	Braasch et al., 2009; Krauss et al., 2014

*Sox10* is a marker gene for chromatophore progenitors (Dutton et al., 2001). Expression was slightly higher in bar than in interbar regions (Figure 5A) and significantly differed between regions (ANOVA: *P* < 0.05). However, we found no significant *sox10* expression variation between bars and interbars (Tukey HSD: *P* = 0.067–0.37; Supplementary Table S2). Expression of the melanophores marker, *mitfa* (Lister et al., 1999; Béjar et al., 2003) was higher within the dark vertical bars than in the adjacent light interbars (Figure 5B and Supplementary Table S2), yet differences were not significant (ANOVA: *P* = 0.19). Similar expression profiles can be also found in *pmel*, a melanophore specific gene important for melanin deposition in melanosomes (Schonthaler et al., 2005). Also here, we find that *pmel is* expressed at a higher yet not significantly higher (ANOVA: *P* = 0.056) level in dark bars compared to interbars (Figure 5C and Supplementary Table S2). The gene *slc24a5*, a melanosome-specific cation exchanger (Lamason et al., 2005), showed differential expression in some pair-wise comparisons of bar and interbar regions (ANOVA: *P* < 0.01; Tukey HSD: *P* = 0.002–0.18; Figure 5D and Supplementary Table S2). The two melanophore-specific genes that express melanogenic enzymes and are essential for the production of melanin, *tyr* (Hidehito et al., 1994; Camp and Lardelli, 2001) and *tyrp1a* (Braasch et al., 2009; Krauss et al., 2014) showed significantly higher expression levels in bars (ANOVA: both *P* < 0.001; Tukey HSD: all *P* < 0.01; Figures 5E,F and Supplementary Table S2).

In order to compare the distribution of iridophores and xanthophores, we used the iridophore lineage-specific marker *ltk* (Lopes et al., 2008) and the xanthophore marker *csf1ra* (Parichy and Turner, 2003). Notably, both *ltk* and *csf1ra* were expressed at similar levels across the differently pigmented bar and interbar regions (Figures 5G,H and Supplementary Table S2). This is in support of a rather homogenous distribution of iridophores and xanthophores across the trunk.

We also examined the gene expression of Agouti family genes (namely *Asip*/*asip1* across all vertebrates and *agrp2* in cichlid fishes) and melanocortin receptors, as they have been previously implicated in pigmentation. Two proteins of the Agouti family, Asip1 and Agrp2, likely act as the antagonists for the melanocortin receptors Mc1r and/or Mc5r in teleost skin (Cal et al., 2017). As previous studies suggest, *asip1* is a key regulator of dorso-ventral countershading, presumably by regulating melanophore number (Ceinos et al., 2015; Cal et al., 2017, 2019). Interestingly we also found significant variation along the anterior-posterior axis (ANOVA: *P* < 0.01) with *asip1* being expressed significantly higher in some pair-wise *post hoc* comparisons between bars and interbars (Tukey HSD: *P* = 0.0016–0.8222; Figure 5I and Supplementary Table S2).

Previous work in cichlids showed that *agrp2* regulates presence/absence of stripe patterns while not contributing to shaping the pigment pattern itself through expression variation across the skin (Kratochwil et al., 2018). In contrast to results from the Lake Victoria species *Pundamilia nyererei*, we found significant differences between skin regions (ANOVA: *P* = 0.041; Figure 5J and Supplementary Table S2). However, expression mainly differed between anterior and posterior regions (Tukey HSD between ib1 and vb2: *P* < 0.05) and not consistently between bars and interbars (Tukey HSD: *P* = 0.046–0.895) (Supplementary Table S2).

Mc1r and Mc5r are two receptors antagonized by Agrp2 and/or Asip1 signaling and *mc1r* and *mc5r* have been shown to be expressed in chromatophores. The expression of *mc1r* has been reported in skin melanophores of zebrafish, barfin, and Japanese flounder (Kobayashi et al., 2010, 2012b; Higdon et al., 2013), xanthophores of goldfish (Kobayashi et al., 2011) and iridophores of zebrafish (Higdon et al., 2013). The expression of *mc5r* was detected in melanophores and xanthophores of flatfish (Kobayashi et al., 2010, 2012b). Here we found no significant expression differences for *mc1r* (ANOVA: *P* = 0.296) and *mc5r* (ANOVA: *P* = 0.764) which suggest that the melanocortin receptors may not contribute to shaping the vertical bar pattern (Figures 5K,L and Supplementary Table S2).

## Discussion

### Melanic Pattern Development: *H. latifasciatus* vs. Other Teleosts

Most previous investigations of teleost pigment pattern formation focused on the horizontal stripe patterns of the model teleost *Danio rerio*, the zebrafish. In zebrafish, melanoblasts, the progenitors of melanophores, migrate from the dorsal neuroectodermal margin along nerve fibers between the myotomes (Dooley et al., 2013). After they settled at their final position, melanoblasts differentiate into melanophores and accumulate melanin to form the dark horizontal stripes that gave the zebrafish its name.

In the haplochromine cichlid *H. latifasciatus* [divergence time with zebrafish approximately 220 million years (Hughes et al., 2018)] color pattern formation starts with the development of four melanophore clusters that arise in the dorsal rim of the trunk (Figures 1B–D). The dorsal clusters initiate the formation of bar patterns later in development, by spreading dorsally into the dorsal fin and ventrally forming bars on the trunk. The third vertical bar (vb3) fuses with the lateral melanophore cluster (Figures 1C,D,I–P and Supplementary Figure S1). In contrast to the zebrafish where adult color pattern develops indirectly during a post-embryonic metamorphic phase (Parichy et al., 2009), the vertical bars in *H. latifasciatus* develop directly and are already visible at a time when the larvae are still feeding from their yolk. This is in line with previous reports on the direct development of other morphological traits of African cichlid fishes (Woltering et al., 2018).

By tracking the behavior of individual melanophores we could observe how new melanophores form over a period of 1–2 days (Figure 2 and Supplementary Figure S1). It is unclear how the melanoblasts reach this position. However, it is likely that they migrate dorsally from the neuroectodermal margin and then ventrally within the skin, possibly explaining the gradual expansion of bars from dorsal to ventral. This is interesting, because in zebrafish clones of single pigment cell progenitor cells have been shown to mainly spread dorso-ventrally (Singh and Nusslein-Volhard, 2015; Nüsslein-Volhard and Singh, 2017). It is possible that vertical bars are prepatterned at the progenitor level, potentially already at the level of the dorsal neuroectodermal ridge. Position and size of bars could be driven by anterior-posterior differences in progenitor number or transcriptional identity that later influence proliferation and differentiation within the dermis. It is also possible that migratory routes, environmental factors along the migratory pathways and in the skin as well as cell-cell interactions including reaction-diffusion systems contribute to or constitute the basis of this process. The anterior-posterior sequence of bar formation could be a consequence of the anterior-posterior sequence of somitogenesis. Anterior melanoblast would hereby first receive guidance cues from dermamyotome, sclerotome, and ermerging dermis. Anterior neural-crest cells including pigment cell precursors would therefore migrate first as previously described (Bronner and LeDouarin, 2012).

**FIGURE 6:**
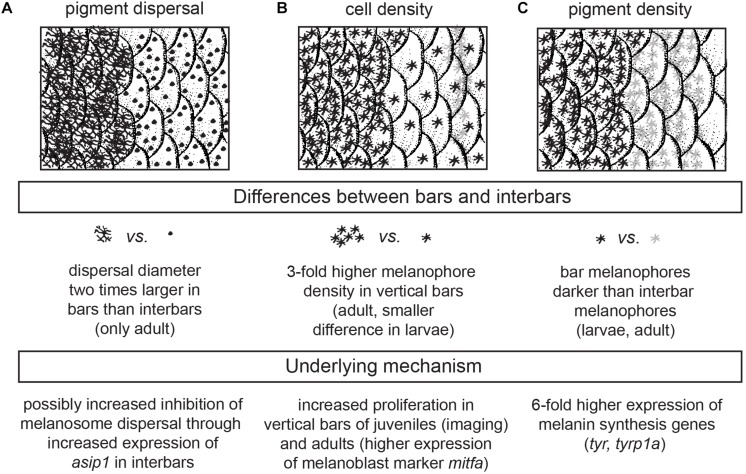
Cellular and genetic mechanism underlying bar pattern formation in *H. latifasciatus*. **(A–C)** The contrast between bars and interbars is driven by three molecularly likely independent mechanisms: melanosome dispersal **(A)**, density of melanophores **(B)** and the darkness of individual melanophores **(C)**.

As soon as melanophores are visible in the skin they do not migrate anymore and grow to the full size within 1–2 days. Hereby the cell-size increases, new dendritic arbors form and melanin levels visibly increase (Figure 2 and Supplementary Figure S1). Therefore, the appearance of the bar pattern is mainly driven by an increase in cell density in the developing bar region as well as changing cellular characteristics including increased melanosome dispersal and melanin production (Figures 2, 6 and Supplementary Figure S1).

As one of the most common pigment patterns in haplochromine cichlids, vertical melanic bars can vary in contrast and number, both between as well as within species (Witte et al., 1976; Kocher et al., 1993; Seehausen et al., 1999). However, compared to most other bared haplochromine cichlids, the vertical bars in *H. latifasciatus* are fixed in number (mostly four and in some strains five) and are relatively thick. Our study shows that vertical bar number remains constant over the course of development in *H. latifasciatus* (Figures 1F–R, 2D–M and Supplementary Figure S1), which contrasts with a recent study on the Lake Malawi cichlid *Copadichromis azureus*, a species with unfixed bar number and thinner bars (Hendrick et al., 2019). In *C. azureus*, vertical bars split during development resulting in an increase of bar number (Hendrick et al., 2019). However, both processes are not driven by rearrangement of excisting pigment cells, but both broadening of bars in *H. latifasciatus* and increase in bar number in *C. azureus* is driven by the formation of new melanophores.

### Cellular Correlates of Bar Patterns and Underlying Mechanism

Comparisons of juvenile and adult patterns largely demonstrate that the juvenile patterns bear already most cellular and morphological characteristics of adult patterns. Vertical bars have a higher density of melanophores and melanophores are also evidently darker in the melanic regions (Figures 4E,F, 6). In contrast to juveniles, pigments are more dispersed in adult bar melanophores. Xanthophores show no clear differences, yet reliable quantification was only partially possible due to the high density of melanophores.

Gene expression analyses of known pigmentation genes gave complementary information as well as mechanistic insights on how the spatial variation of chromatophore properties and densities are archieved (Figures 5, 6). Clearly, melanophore-specific genes such as *tyr*, *tyrp1a*, but also *slc24a5* are differentially expressed between bars and interbars (Figures 5D–F and Supplementary Table S2). All three genes are essential for melanin production. The two tyrosinases are directly involved in the synthesis of melanin, *slc24a5*, a potassium-dependent sodium/calcium exchanger is thought to modulate melanosomal pH, which is a crucial parameter for melanin synthesis (Ginger et al., 2008). Also *pmel*, the premelanosome protein, another melanophore-specific gene is slightly, yet not significantly, upregulated in bar regions compared to interbar regions (Figure 5C and Supplementary Table S2). Overall, expression data show a sixfold higher expression of tyrosinases in bars providing an explanation for the darker appearance of bar melanophores (Figure 6 and Supplementary Table S2). Even, if we standardize the differential expression by the increased cell-number or the melanophore-lineage marker *mitfa* we still find a 2–4.5-fold (Supplementary Figure S7 and Supplementary Tables S3, S4) increase of tyrosinase expression and therefore likely melanin synthesis.

Although we did not find significant differential expression of the transcription factors *sox10* and *mitfa* (Figures 5A,B), both crucial factors for melanophore differentiation, expression is slightly elevated in bar regions. As *mitfa* expression has been reported to be only weak in differentiated melanophores (Johnson et al., 2011), this result might indicate an elevated number of melanoblasts. Although this would have to be analyzed in detail, it would explain the increased number of newly forming melanophores in the bars of juveniles (Figure 2 and Supplementary Figure S1) and consequently an increase in melanophore density.

Additionally, we used a marker gene for the xanthophore lineage (*csf1ra*) and the iridophore lineage (*ltk*). None of them showed any expression differences (Figures 5G,H), suggesting that there is no obvious difference in xanthophore and iridophore number. Therefore, melanophores seem to be the main cell type that clearly differs both in number and expression of marker genes between bar and interbar regions. Although there is some indication of a decreased number and coverage of xanthophores in the bar regions, this analysis was greatly hindered by the dense melanophore coverage. Use of mutant lines as for example of the *oca2* gene that will likely not affect the pattering of xanthophores and iridophores (Kratochwil et al., 2019b) would be a possibility to better assess this question. Still, this result is somewhat surprising as cell-cell interactions between xanthophores, melanophores and iridophores seem to have a completely different dynamic in cichlids compared to zebrafish, where these cell types are spatially segregated (Mahalwar et al., 2014; Patterson et al., 2014; Singh et al., 2014; Eom and Parichy, 2017). In zebrafish, especially xanthophores and melanophores are almost mutually exclusive, with a decreased cell number of xanthophores in the melanic stripes. No evidence of such a strong antagonistic interaction could be found in *H. latifasciatus*. In xanthophore morphology though, zebrafish and *H. latifasciatus* have some shared featured: xanthophores in melanic regions are more irregular and seem smaller and have fewer and not as evenly distributed dendritic arbors (Figures 3, 4, Supplementary Figure S5, and Supplementary Table S1), although we did not find significant differences in xanthophore diameter (Figures 4I,J and Supplementary Table S1).

Our result on the expression of *mc1r* and *mc5r* shows that the melanocortin receptors likely play no role in shaping the vertical bar patterns in *H. latifasciatus* (Figures 5K,L). The antagonists *asip1* and *agrp2* show significant differences in expression (Figures 5I,J and Supplementary Table S2). The gene *agrp2* has been shown to inhibit the formation of horizontal stripe patterns: species with high *agrp2* expression lack any horizontal stripe patterns, while species with horizontal stripes have generally low expression of *agrp2* in skin. Yet, spatial variation in *agrp2* expression does not seem to be necessary for the formation of stripes (Kratochwil et al., 2018). On the other hand, a gradient in *agrp2* expression along anterio-posterior axis could be found in both *H. latifasciatus* with significant difference from this study (Figure 5J and Supplementary Table S2) and *P. nyererei* without significant difference from our previous study (Kratochwil et al., 2018). However, based on our previous work that showed no changes in bar patterns in a knockout of *agrp2* in *P. nyererei* (Kratochwil et al., 2018) or association with bar presence and absence (Kratochwil et al., 2019a), a role in vertical bar formation seems rather unlikely.

The variation in *asip1* expression is intriguing. The melanocortin signaling antagonist *asip1* has higher expression in interbars (twofold difference; Supplementary Table S2) with a particularly high expression in the first interbar. This is surprising as *asip1* (and also the tetrapod homolog *Asip*) was previously known to vary along the dorso-ventral axis where it is involved in generating the dorso-ventral countershading that can be seen in many vertebrates (Manceau et al., 2011; Cal et al., 2017, 2019; Haupaix et al., 2018; Kratochwil, 2019). Here we observed variation along the anterio-posterior axis (Figure 5I and Supplementary Table S2), suggesting that gene expression differences of *asip1* might contribute to variation in pigmentation on both axes. As Asip1 acts as Mc1r/Mc5r antagonist, one of the responses of decreased melanocortin signaling would be an increased aggregation of melanosomes. This would be in line with the significant difference in dispersion diameter we observe between bars and interbars. In interbars the diameter is on average 2.8 times smaller (Figures 4C,D and Supplementary Table S1), contributing to the lighter appearance of the interbar region (Figure 6). Yet the variation of *asip1* expression serves only as a partial explanation as (a) the expression level in int2 is similar to the adjacent bars and (b) the dispersion/aggregation states of melanophores vary greatly within bars and interbars as well as between individuals. Still, based on these findings, it might be an important take-home-message to consider functions of asip1 beyond dorso-ventral patterning (i.e., countershading).

## Conclusion

In summary, we investigated the formation of the vertical bar color pattern of *H. latifasciatus* – a member of the phenotypically diverse East African haplochromine cichlid fish radiations – during embryonic and larval development, how the macroscopic patterns is formed through variation in pigment cell distribution and properties, and how this variation links to known coloration gene expression. Our work provides novel insights into the molecular and cellular properties that contribute to the formation of color patterns in this famously diverse family of fish. More specifically we demonstrate that bar pattern formation is facilitated by three molecularly likely independent mechanisms: increased melanosome dispersal (controlled by melanosome migration along the cytoskeleton), density of melanophores (controlled by proliferation in progenitors) and melanin synthesis (controlled by melanin synthesis pathways and melanosome micro-environment) (Figure 6). *H. latifasciatus* with its – as we describe here – morphologically and transcriptomically well defined bar and interbar regions that can be tracked throughout development at a cellular level provides a unique system to further understand the molecular and cellular underpinnings of color patterns. However, further investigations on the development of vertical bars by assaying gene expression using RNA-seq, *in situ* hybridization and immunohistochemistry as well as comparative analyses with closely related species will be particularly informative for further understanding the molecular mechanisms underlying vertical bar formation. Cichlids with a rich and expanding repertoire of experimental approaches including hybrid crosses, transgenesis and CRISPR-Cas9 genome editing (Kratochwil and Meyer, 2015; Juntti, 2019) will make this species an excellent choice for further investigating the causal genetic variants, genes and molecular mechanisms that influenced evolution of and variation in vertical bar pattern formation.

## Materials and Methods

### Fish Husbandry and Embryo Culture

*H. latifasciatus* were kept in groups of 10–25 individuals. Fertilized eggs were removed from the gravid females as early as possible. The larvae and juvenile fish were then raised in egg incubators (ZET-E55, Ziss aqua) at room temperature. At 5 days post-fertilization (dpf) three larvae were separated from their siblings and each of them was raised in a single incubator to be able to keep track of a single individual in the following weeks. The hatching fish larvae were provided with nutrients by their yolk sac which lasts for ∼14 days. After 14 dpf the larvae were fed on *Artemia nauplii* twice a day. Experiments were performed in accordance with the rules of the animal research facility of the University of Konstanz, Germany and with the permission of the animal care committee (Regierungspräsidium) Freiburg, Germany (G18/60 and T16/13).

### Fish Larvae Photography and Analysis

Photographs of *H. latifasciatus* embryos and larvae were captured with a stereomicroscope (Leica MZ10F) with a Leica DMC2900 color camera. Fish were first anesthetized with 0.04% tricaine (MS-222, Sigma-Aldrich). Images were taken as previously described (Kratochwil et al., 2015; Kratochwil et al., 2017). In order to capture the color pattern development of *H. latifasciatus*, photographs were taken from 7 to 21 dpf of three individuals.

For quantitative analyses, a comparable area including vb2 and vb3 of photographs of three individuals from stages between 9 and 21 dpf were put together, aligned (using the anterior dorsal fin as landmark), transformed into a black-and-white image and quantified in three different ways using Fiji (Schindelin et al., 2012). Melanosome dispersal was estimated by measuring the diameter of the minimally sized circle that encloses all melanic parts of a melanophore (minimal enclosing circle). It is therefore affected both by the melanophore size and the dispersal/aggregation state of melanosomes within the melanophores. The relative gray value was measured by taking the mean gray value of the same circle and dividing it by the mean of all melanophore gray value measurements of the same individual and stage. This relative value was used to account for differences between images and stages. Melanophore density was calculated from manual melanophore counts. As the position of the bars would shift as the fish is growing, we corrected the position values by the growth of the individual (size of individual at X dph/size of individual at 9 dph). The values were plotted using non-parametric regression (locally weighted scatterplot smoothing; LOWESS) in R (f parameter: 0.1). For the density calculation the area was split into ten equally-sized zones. To track individual melanophores we took images of the same individual on five consecutive days (from 9 to 13 dpf; Figures 2D–H). The identity of single melanophores across stages was estimated using overlays of the images from multiple days.

### Image Acquisition in Adult Fish

Three female individuals (standard length ∼12 cm) were examined for pigment quantification. We only quantified the chromatophores in females as the vertical bar pattern does not differ between sexes, we found more variation in red coloration in males (Figures 1Q,R). For each fish, in total 156 photos were obtained from scales and skin with scales removed (referred to as skin). Firstly, we separated the two flanks with scales still attached to skin. Scales from study regions were then carefully removed from the left flank and kept separately in Ringer’s solution (6.5 g/L NaCl, 0.25 g/L KCl, 0.3 g/L CaCl_2_ and 0.2 g/L NaHCO_3_) at 4°C before imaging. Five light microscope photographs were taken on each melanic and non-melanic region (the two melanic bar regions vb2 and vb3, and the interbar ib1 which anterior of vb2 and ib3 which between vb2 and vb3) from dorsal to ventral with a Leica MZ10F stereomicroscope equipped with a Leica DMC2900 color camera. Four photos were taken also along the dorsal-ventral axis on both region using Lecia MZ10F equipped with a Leica DFC 3000G black-and-white camera with GFP filter to image the auto-fluoresce of adult xanthophores (Kelsh et al., 1996; Guyader and Jesuthasan, 2002). For each pigmented region, ten scales were imaged using a Leica DMC2900 camera on Leica DM6B upright microscope. Leica Application Suite X software was used to capture the photos using the same setting. To count the number of melanophores, the right flank including both scales and skin tissue was treated with 10 mg/ml epinephrine (SIGMA-ALDRICH) for 20 min at room temperature to aggregate the melanosomes and thereby permit robust cell number quantifications (Figures 3B,F,K,N). After epinephrine treatment, tissue was washed by Phosphate Buffered Saline (PBS, pH7.4) three times and then kept in 4% Paraformaldehyde (PFA) in PBS. Epinephrine-treated scales were removed from skin and photos were taken for both skin and scales as described above. Fluorescence images were not taken as this treatment caused a high autofluorescence background.

### Image and Data Analysis of Adult Patterns

All photos were analyzed with the image analysis software Fiji (Schindelin et al., 2012). At the beginning of the analysis we manually adjusted the color threshold to obtain reliable quantification of the bright field photos from non-treated skin and scales. Using this setting we executed the “Analyze Particles” function to obtain the melanophore coverage. For 20 randomly selected melanophores from each skin image and 10 melanophores from each scale image we measured the dispersal diameter of melanin covered parts of the melanophores (as described above). For xanthophore coverage we used the same approach. The dispersal diameter of the pigment filled part of 20 xanthophores of each skin image and 10 xanthophores of each scale image was measured from each photograph. The number of melanophore and xanthophore was counted from epinephrine-treated skin and scale specimens. Single xanthophores on scales could be observed easily by fluorescence microscopy, while not all boundaries of xanthophores in the skin could be easily identified as the cells often overlapped. Therefore, we were able to count the xanthophore number in scales but not in the skin dissections, while dispersal diameter measurements for xanthophores was possible on both scales and skin. Xanthophores and (what likely are erythrophores) was treated as the same cell type. Several studies show that erythrophores also exist in cichlids (Chen et al., 2014, 2015). However, vesicles containing pteridine and carotenoids could be found in the same cells, in which case the overall color depends on the ratio of red and yellow pigments (Matsumoto, 1965; Bagnara, 1966). Hence, the distinction between xanthophores and erythrophores is not always clear. Therefore, we classified yellow/orange/red colored cells all as xanthophores. Although we could identify iridophores in both dark bars and light interbars before and after epinephrine treatment (Figures 3I,K,L,N), iridophores were mostly below or above melanophores hindering reliable measurements. We, therefore, used gene expression of the iridophore lineage marker gene *ltk* (Lopes et al., 2008).

### RNA Extraction

To compare the expression of coloration and pigment genes between melanic bars and interbars regions, we sampled the whole melanic and non-melanic skin region. Skin tissue was dissected and kept in RNAlater (Invitrogen) at 4°C overnight and then transferred to −20°C for long-term storage. RNAlater was removed prior to homogenization. Skin samples and the appropriate amount of TRIzol (Invitrogen) (1 ml TRIzol per 100 mg sample) were homogenized in 2 ml Lysing Matrix A tube (MP Biomedicals) using FastPrep-24 Classic Instrument (MP Biomedicals). RNA was extracted according to the manufacturer’s recommendations (Invitrogen) with an additional wash step by 75% Ethanol. Subsequent purification and on-column DNase treatment were performed with the RNeasy Mini Kit (Qiagen) and RNase-Free DNase Set (Qiagen). Following extraction and purification, RNA was quantified using Qubit RNA BR Assay Kit (Invitrogen) with Qubit Fluorometer (Life Technologies).

### Quantitative Real-Time PCR (RT-qPCR)

Gene expression analyses were performed on two melanic (vb2 and vb3) and two non-melanic (ib1 and ib2) skin regions. First strand cDNA was synthesized by using 1μg of total RNA with a GoScript Reverse Transcription System (Promega). qPCRs were performed with 2 μl of 100 μl synthesized first strand cDNA that was diluted ten times from 20 μl of initial reaction volume as a template, 10pmol of each forward primer and reverse primer, and GoTaq qPCR Master Mix (Promega) with nuclease-free water to make the final volume of 20 μl in a 96-well plate. Twelve genes were processed to examine the expression level including *sox10, mitfa, csf1ra, ltk, pmel, slc24a5, tyr, tyrp1a, asip1, agrp2, mc1r, mc5r* (Table 1). Primers are listed in Supplementary Table S5. We used 40 cycles of amplification on a CFX96 Real-Time PCR Detection System (Bio-Rad). The amplification program was: initial denaturation at 95°C for 10 min, 40 cycles of 95°C for 20 s, 60°C for 60 s. At the end of the cycles, melting curve of the products was verified for the specificity of PCR products. Only samples with one peak in the melting curves were processed to analyses. We assayed gene expression in triplicate for each sample and normalized the data using the reference genes β*-actin* and *gapdh*. Ct values were defined as the point at which fluorescence crossed a threshold (R_*Ct*_) adjusted manually to be the point at which fluorescence rose above the background level. Next, we compared the relative expression between samples using the 2^–ΔΔCT^ method (Nolan et al., 2006). For group comparisons, we used ANOVA followed by Tukey’s HSD. All statistical tests were performed in R (R Development Core Team, 2019).

## Data Availability Statement

The raw data supporting the conclusions of this article will be made available by the authors, without undue reservation, to any qualified researcher.

## Ethics Statement

The animal study was reviewed and approved by the Animal Care Committee (Regierungspräsidium) Freiburg, Germany.

## Author Contributions

YL: investigation, visualization, methodology, formal analysis, funding acquisition, validation, writing – original draft, and writing – review and editing. JG: investigation. AM: supervision, funding acquisition, and writing – review and editing. CK: conceptualization, resources, formal analysis, supervision, funding acquisition, visualization, methodology, writing – original draft, project administration, and writing – review and editing.

## Conflict of Interest

The authors declare that the research was conducted in the absence of any commercial or financial relationships that could be construed as a potential conflict of interest.

## References

[B1] Ahi E. P. Sefc K. M. (2017). Anterior-posterior gene expression differences in three Lake Malawi cichlid fishes with variation in body stripe orientation. *PeerJ* 5:e4080. 10.7717/peerj.4080 29158996 PMC5695249

[B2] Albertson R. C. Powder K. E. Hu Y. Coyle K. P. Roberts R. B. Parsons K. J. (2014). Genetic basis of continuous variation in the levels and modular inheritance of pigmentation in cichlid fishes. *Mol. Ecol.* 23 5135–5150. 10.1111/mec.12900 25156298 PMC4238941

[B3] Bagnara J. T. (1966). “Cytology and cytophysiology of non-melanophore pigment cells,” in *International Review of Cytology*, Ed. Jeon K. W., (Amsterdam: Elsevier), 173–205. 10.1016/s0074-7696(08)60801-3 5337298

[B4] Béjar J. Hong Y. Schartl M. (2003). Mitf expression is sufficient to direct differentiation of medaka blastula derived stem cells to melanocytes. *Development* 130 6545–6553. 10.1242/dev.00872 14660543

[B5] Braasch I. Liedtke D. Volff J. N. Schartl M. (2009). Pigmentary function and evolution of tyrp1 gene duplicates in fish. *Pigment Cell Melanoma Res.* 22 839–850. 10.1111/j.1755-148x.2009.00614.x 19659755

[B6] Bronner M. E. LeDouarin N. M. (2012). Development and evolution of the neural crest: an overview. *Dev. Biol.* 366 2–9. 10.1016/j.ydbio.2011.12.042 22230617 PMC3351559

[B7] Burton D. Burton M. (2017). *Essential Fish Biology: Diversity, Structure, and Function.* Oxford: Oxford University Press.

[B8] Cal L. Megias M. Cerda-Reverter J. M. Postlethwait J. H. Braasch I. Rotllant J. (2017). BAC Recombineering of the Agouti Loci from Spotted Gar and Zebrafish Reveals the Evolutionary Ancestry of Dorsal-Ventral Pigment Asymmetry in Fish. *J. Exp Zool. B Mol. Dev. Evol.* 328 697–708. 10.1002/jez.b.22748 28544213 PMC5653409

[B9] Cal L. Suarez-Bregua P. Comesana P. Owen J. Braasch I. Kelsh R. (2019). Countershading in zebrafish results from an Asip1 controlled dorsoventral gradient of pigment cell differentiation. *Sci. Rep.* 9:3449. 10.1038/s41598-019-40251-z 30837630 PMC6401153

[B10] Camp E. Lardelli M. (2001). Tyrosinase gene expression in zebrafish embryos. *Dev. Genes Evol.* 211 150–153. 10.1007/s004270000125 11455427

[B11] Ceinos R. M. Guillot R. Kelsh R. N. Cerda-Reverter J. M. Rotllant J. (2015). Pigment patterns in adult fish result from superimposition of two largely independent pigmentation mechanisms. *Pigment Cell Melanoma Res.* 28 196–209. 10.1111/pcmr.12335 25469713

[B12] Cerda-Reverter J. M. Haitina T. Schioth H. B. Peter R. E. (2005). Gene structure of the goldfish agouti-signaling protein: a putative role in the dorsal-ventral pigment pattern of fish. *Endocrinology* 146 1597–1610. 10.1210/en.2004-1346 15591139

[B13] Chakraborty A. K. Platt J. T. Kim K. K. Kwon B. S. Bennett D. C. Pawelek J. M. (1996). Polymerization of 5,6-dihydroxyindole-2-carboxylic acid to melanin by the pmel 17/silver locus protein. *Eur. J. Biochem.* 236 180–188. 10.1111/j.1432-1033.1996.t01-1-00180.x 8617263

[B14] Chen S. C. Hornsby M. A. Robertson R. M. Hawryshyn C. W. (2014). The influence of chromatic background on the photosensitivity of tilapia erythrophores. *Biol. Open* 3 117–120. 10.1242/bio.20146742 24414206 PMC3925314

[B15] Chen S. C. Xiao C. Troje N. F. Robertson R. M. Hawryshyn C. W. (2015). Functional characterisation of the chromatically antagonistic photosensitive mechanism of erythrophores in the tilapia *Oreochromis niloticus*. *J. Exp. Biol.* 218 748–756. 10.1242/jeb.106831 25573822

[B16] Dooley C. M. Mongera A. Walderich B. Nusslein-Volhard C. (2013). On the embryonic origin of adult melanophores: the role of ErbB and Kit signalling in establishing melanophore stem cells in zebrafish. *Development* 140 1003–1013. 10.1242/dev.087007 23364329

[B17] Du J. Miller A. J. Widlund H. R. Horstmann M. A. Ramaswamy S. Fisher D. E. (2003). MLANA/MART1 and SILV/PMEL17/GP100 are transcriptionally regulated by MITF in melanocytes and melanoma. *Am. J. Pathol.* 163 333–343. 10.1016/s0002-9440(10)63657-7 12819038 PMC1868174

[B18] Dutton K. A. Pauliny A. Lopes S. S. Elworthy S. Carney T. J. Rauch J. (2001). Zebrafish colourless encodes sox10 and specifies non-ectomesenchymal neural crest fates. *Development* 128 4113–4125. 11684650 10.1242/dev.128.21.4113

[B19] Elmer K. R. Lehtonen T. K. Meyer A. (2009). Color assortative mating contributes to sympatric divergence of neotropical cichlid fish. *Evolution* 63 2750–2757. 10.1111/j.1558-5646.2009.00736.x 19490078

[B20] Elworthy S. Lister J. A. Carney T. J. Raible D. W. Kelsh R. N. (2003). Transcriptional regulation of mitfa accounts for the sox10 requirement in zebrafish melanophore development. *Development* 130 2809–2818. 10.1242/dev.00461 12736222

[B21] Eom D. S. Parichy D. M. (2017). A macrophage relay for long-distance signaling during postembryonic tissue remodeling. *Science* 355 1317–1320. 10.1126/science.aal2745 28209639 PMC5836293

[B22] Ginger R. S. Askew S. E. Ogborne R. M. Wilson S. Ferdinando D. Dadd T. (2008). SLC24A5 encodes a trans-Golgi network protein with potassium-dependent sodium-calcium exchange activity that regulates human epidermal melanogenesis. *J. Biol. Chem.* 283 5486–5495. 10.1074/jbc.m707521200 18166528

[B23] Greenwood P. H. (1974). Cichlid fishes of Lake Victoria, East Africa: the biology and evolution of a species flock. *Bull. Br. Mus. Nat. Hist. Zool. Suppl.* 6 1–134.

[B24] Guillot R. Ceinos R. M. Cal R. Rotllant J. Cerda-Reverter J. M. (2012). Transient ectopic overexpression of agouti-signalling protein 1 (asip1) induces pigment anomalies in flatfish. *PLoS One* 7:e48526. 10.1371/journal.pone.0048526 23251332 PMC3519472

[B25] Guyader S. L. Jesuthasan S. (2002). Analysis of xanthophore and pterinosome biogenesis in zebrafish using methylene blue and pteridine autofluorescence. *Pigment Cell Res.* 15 27–31. 10.1034/j.1600-0749.2002.00045.x 11837453

[B26] Haupaix N. Curantz C. Bailleul R. Beck S. Robic A. Manceau M. (2018). The periodic coloration in birds forms through a prepattern of somite origin. *Science* 361:eaar4777. 10.1126/science.aar4777 30237324

[B27] Hemingson C. R. Cowman P. F. Hodge J. R. Bellwood D. R. (2019). Colour pattern divergence in reef fish species is rapid and driven by both range overlap and symmetry. *Ecol. Lett.* 22 190–199. 10.1111/ele.13180 30467938

[B28] Hendrick L. A. Carter G. A. Hilbrands E. H. Heubel B. P. Schilling T. F. Le Pabic P. (2019). Bar, stripe and spot development in sand-dwelling cichlids from Lake Malawi. *Evodevo* 10:18. 10.1186/s13227-019-0132-7 31417669 PMC6691528

[B29] Henning F. Meyer A. (2012). Eggspot number and sexual selection in the cichlid fish *Astatotilapia burtoni*. *PLoS One* 7:e43695. 10.1371/journal.pone.0043695 22937082 PMC3427294

[B30] Hidehito I. Yoshitaka B. Akihiko K. Hiroshi H. (1994). Expression of the tyrosinase-encoding gene in a colorless melanophore mutant of the medaka fish *Oryzias latipes*. *Gene* 150 319–324. 10.1016/0378-1119(94)90445-6 7821799

[B31] Higdon C. W. Mitra R. D. Johnson S. L. (2013). Gene expression analysis of zebrafish melanocytes, iridophores, and retinal pigmented epithelium reveals indicators of biological function and developmental origin. *PLoS One* 8:e67801. 10.1371/journal.pone.0067801 23874447 PMC3706446

[B32] Hou L. Arnheiter H. Pavan W. J. (2006). Interspecies difference in the regulation of melanocyte development by SOX10 and MITF. *Proc. Natl. Acad. Sci. U.S.A.* 103 9081–9085. 10.1073/pnas.0603114103 16757562 PMC1482569

[B33] Hughes L. C. Orti G. Huang Y. Sun Y. Baldwin C. C. Thompson A. W. (2018). Comprehensive phylogeny of ray-finned fishes (*Actinopterygii*) based on transcriptomic and genomic data. *Proc. Natl. Acad. Sci. U.S.A.* 115 6249–6254. 10.1073/pnas.1719358115 29760103 PMC6004478

[B34] Irion U. Nüsslein-Volhard C. (2019). The identification of genes involved in the evolution of color patterns in fish. *Curr. Opin. Genet. Dev.* 57 31–38. 10.1016/j.gde.2019.07.002 31421397 PMC6838669

[B35] Johnson S. L. Nguyen A. N. Lister J. A. (2011). Mitfa is required at multiple stages of melanocyte differentiation but not to establish the melanocyte stem cell. *Dev. Biol.* 350 405–413. 10.1016/j.ydbio.2010.12.004 21146516 PMC3040983

[B36] Juntti S. (2019). The future of gene-guided neuroscience research in non-traditional model organisms. *Brain Behav. Evol.* 93 108–121. 10.1159/000500072 31416064

[B37] Kelsh R. N. Brand M. Jiang Y. J. Heisenberg C. P. Lin S. Haffter P. (1996). Zebrafish pigmentation mutations and the processes of neural crest development. *Development* 123 369–389. 9007256 10.1242/dev.123.1.369

[B38] Kobayashi Y. Chiba H. Mizusawa K. Suzuki N. Cerda-Reverter J. M. Takahashi A. (2011). Pigment-dispersing activities and cortisol-releasing activities of melanocortins and their receptors in xanthophores and head kidneys of the goldfish *Carassius auratus*. *Gen. Comp. Endocrinol.* 173 438–446. 10.1016/j.ygcen.2011.06.019 21784075

[B39] Kobayashi Y. Mizusawa K. Chiba H. Tagawa M. Takahashi A. (2012a). Further evidence on acetylation-induced inhibition of the pigment-dispersing activity of α-melanocyte-stimulating hormone. *Gen. Comp. Endocrinol.* 176 9–17. 10.1016/j.ygcen.2011.12.001 22197208

[B40] Kobayashi Y. Mizusawa K. Saito Y. Takahashi A. (2012b). Melanocortin systems on pigment dispersion in fish chromatophores. *Front. Endocrinol.* 3:9 10.3389/fendo.2012.00009 PMC3355986 22649405

[B41] Kobayashi Y. Tsuchiya K. Yamanome T. Schioth H. B. Takahashi A. (2010). Differential expressions of melanocortin receptor subtypes in melanophores and xanthophores of barfin flounder. *Gen. Comp. Endocrinol.* 168 133–142. 10.1016/j.ygcen.2010.04.017 20417636

[B42] Kocher T. D. Conroy J. A. Mckaye K. R. Stauffer J. R. (1993). Similar morphologies of cichlid fish in Lakes Tanganyika and Malawi are due to convergence. *Mol. Phylogenet. Evol.* 2 158–165. 10.1006/mpev.1993.1016 8025722

[B43] Korner A. Pawelek J. (1982). Mammalian tyrosinase catalyzes three reactions in the biosynthesis of melanin. *Science* 217 1163–1165. 10.1126/science.6810464 6810464

[B44] Kratochwil C. F. (2019). Molecular mechanisms of convergent color pattern evolution. *Zoology* 134 66–68. 10.1016/j.zool.2019.04.004 31146908

[B45] Kratochwil C. F. Liang Y. Gerwin J. Woltering J. M. Urban S. Henning F. (2018). Agouti-related peptide 2 facilitates convergent evolution of stripe patterns across cichlid fish radiations. *Science* 362 457–460. 10.1126/science.aao6809 30361373

[B46] Kratochwil C. F. Liang Y. Urban S. Torres-Dowdall J. Meyer A. (2019a). Evolutionary dynamics of structural variation at a key locus for color pattern diversification in cichlid fishes. *Genome Biol. Evol.* 11 3452–3465. 10.1093/gbe/evz261 31821504 PMC6916709

[B47] Kratochwil C. F. Urban S. Meyer A. (2019b). Genome of the Malawi golden cichlid fish (*Melanochromis auratus*) reveals exon loss of *oca2* in an amelanistic morph. *Pigment Cell Melanoma Res.* 32 719–723. 10.1111/pcmr.12799 31131985

[B48] Kratochwil C. F. Meyer A. (2015). Closing the genotype-phenotype gap: emerging technologies for evolutionary genetics in ecological model vertebrate systems. *Bioessays* 37 213–226. 10.1002/bies.201400142 25380076

[B49] Kratochwil C. F. Sefton M. M. Liang Y. Meyer A. (2017). Tol2 transposon-mediated transgenesis in the Midas cichlid (*Amphilophus citrinellus*) - towards understanding gene function and regulatory evolution in an ecological model system for rapid phenotypic diversification. *BMC Dev. Biol.* 17:15. 10.1186/s12861-017-0157-x 29169323 PMC5701313

[B50] Kratochwil C. F. Sefton M. M. Meyer A. (2015). Embryonic and larval development in the Midas cichlid fish species flock (*Amphilophus* spp.): a new evo-devo model for the investigation of adaptive novelties and species differences. *BMC Dev. Biol.* 15:12. 10.1186/s12861-015-0061-1 25887993 PMC4352272

[B51] Krauss J. Geiger-Rudolph S. Koch I. Nüsslein-Volhard C. Irion U. (2014). A dominant mutation in tyrp1 A leads to melanophore death in zebrafish. *Pigment Cell Melanoma Res.* 27 827–830. 10.1111/pcmr.12272 24891189

[B52] Kurokawa T. Murashita K. Uji S. (2006). Characterization and tissue distribution of multiple agouti-family genes in pufferfish *Takifugu rubripes*. *Peptides* 27 3165–3175. 10.1016/j.peptides.2006.09.013 17097766

[B53] Lamason R. L. Mohideen M. A. Mest J. R. Wong A. C. Norton H. L. Aros M. C. (2005). SLC24A5, a putative cation exchanger, affects pigmentation in zebrafish and humans. *Science* 310 1782–1786. 10.1126/science.1116238 16357253

[B54] Lister J. A. Robertson C. P. Lepage T. Johnson S. L. Raible D. W. (1999). nacre encodes a zebrafish microphthalmia-related protein that regulates neural-crest-derived pigment cell fate. *Development* 126 3757–3767. 10433906 10.1242/dev.126.17.3757

[B55] Lopes S. S. Yang X. Muller J. Carney T. J. Mcadow A. R. Rauch G. J. (2008). Leukocyte tyrosine kinase functions in pigment cell development. *PLoS Genet.* 4:e1000026. 10.1371/journal.pgen.1000026 18369445 PMC2265441

[B56] Maan M. E. Sefc K. M. (2013). Colour variation in cichlid fish: developmental mechanisms, selective pressures and evolutionary consequences. *Semin. Cell Dev. Biol.* 24 516–528. 10.1016/j.semcdb.2013.05.003 23665150 PMC3778878

[B57] Mahalwar P. Walderich B. Singh A. P. Nusslein-Volhard C. (2014). Local reorganization of xanthophores fine-tunes and colors the striped pattern of zebrafish. *Science* 345 1362–1364. 10.1126/science.1254837 25214630

[B58] Manceau M. Domingues V. S. Mallarino R. Hoekstra H. E. (2011). The developmental role of Agouti in color pattern evolution. *Science* 331 1062–1065. 10.1126/science.1200684 21350176

[B59] Matsumoto J. (1965). Studies on Fine structure and cytochemical properties of erythrophores in Swordtail Xiphophorus Helleri with special reference to their pigment granules (*Pterinosomes*). *J. Cell Biol.* 27 493–504. 10.1083/jcb.27.3.493 5885426 PMC2106771

[B60] Meyer A. Biermann C. H. Orti G. (1993). The phylogenetic position of the zebrafish (*Danio rerio*), a model system in developmental biology: an invitation to the comparative method. *Proc. Biol. Sci.* 252 231–236. 10.1098/rspb.1993.0070 8394584

[B61] Meyer A. Ritchie P. A. Witte K. E. (1995). Predicting developmental processes from evolutionary patterns - a molecular phylogeny of the zebrafish (*Danio-rerio*) and Its Relatives. *Philos. Trans. R. Soc. B Biol. Sci.* 349 103–111. 10.1098/rstb.1995.0096

[B62] Nagao Y. Suzuki T. Shimizu A. Kimura T. Seki R. Adachi T. (2014). Sox5 functions as a fate switch in Medaka pigment cell development. *PLoS Genet.* 10:e1004246. 10.1371/journal.pgen.1004246 24699463 PMC3974636

[B63] Nolan T. Hands R. E. Bustin S. A. (2006). Quantification of mRNA using real-time RT-PCR. *Nat. Protoc.* 1 1559–1582. 10.1038/nprot.2006.236 17406449

[B64] Nüsslein-Volhard C. Singh A. P. (2017). How fish color their skin: a paradigm for development and evolution of adult patterns: multipotency, plasticity, and cell competition regulate proliferation and spreading of pigment cells in zebrafish coloration. *BioEssays* 39:1600231. 10.1002/bies.201600231 28176337

[B65] Parichy D. M. Elizondo M. R. Mills M. G. Gordon T. N. Engeszer R. E. (2009). Normal table of postembryonic zebrafish development: staging by externally visible anatomy of the living fish. *Dev. Dyn.* 238 2975–3015. 10.1002/dvdy.22113 19891001 PMC3030279

[B66] Parichy D. M. Ransom D. G. Paw B. Zon L. I. Johnson S. L. (2000). An orthologue of the kit-related gene fms is required for development of neural crest-derived xanthophores and a subpopulation of adult melanocytes in the zebrafish. *Danio rerio*. *Development* 127 3031–3044. 10862741 10.1242/dev.127.14.3031

[B67] Parichy D. M. Turner J. M. (2003). Temporal and cellular requirements for Fms signaling during zebrafish adult pigment pattern development. *Development* 130 817–833. 10.1242/dev.00307 12538511

[B68] Patterson L. B. Bain E. J. Parichy D. M. (2014). Pigment cell interactions and differential xanthophore recruitment underlying zebrafish stripe reiteration and *Danio* pattern evolution. *Nat. Commun.* 5:5299. 10.1038/ncomms6299 25374113 PMC4224114

[B69] Patterson L. B. Parichy D. M. (2019). Zebrafish pigment pattern formation: insights into the development and evolution of adult form. *Ann. Rev. Genet.* 53 505–530. 10.1146/annurev-genet-112618-043741 31509458

[B70] Prazdnikov D. V. Shkil F. N. (2019). Experimental evidence of the role of heterochrony in evolution of the Mesoamerican cichlids pigment patterns. *Evol. Dev.* 21 3–15. 10.1111/ede.12272 30239104

[B71] R Development Core Team (2019). *R: A Language and Environment for Statistical Computing.* Vienna: R Foundation for Statistical Computing.

[B72] Richardson J. Lundegaard P. R. Reynolds N. L. Dorin J. R. Porteous D. J. Jackson I. J. (2008). mc1r Pathway regulation of zebrafish melanosome dispersion. *Zebrafish* 5 289–295. 10.1089/zeb.2008.0541 19133827

[B73] Roberts R. B. Ser J. R. Kocher T. D. (2009). Sexual conflict resolved by invasion of a novel sex determiner in Lake Malawi cichlid fishes. *Science* 326 998–1001. 10.1126/science.1174705 19797625 PMC3174268

[B74] Roux N. Salis P. Lambert A. Logeux V. Soulat O. Romans P. (2019). Staging and normal table of postembryonic development of the clownfish (*Amphiprion ocellaris*). *Dev. Dyn.* 248 545–568. 10.1002/dvdy.46 31070818 PMC6771578

[B75] Salis P. Roux N. Soulat O. Lecchini D. Laudet V. Frédérich B. (2018). Ontogenetic and phylogenetic simplification during white stripe evolution in clownfishes. *BMC Biol.* 16:90. 10.1186/s12915-018-0559-7 30180844 PMC6123960

[B76] Santos M. E. Baldo L. Gu L. Boileau N. Musilova Z. Salzburger W. (2016). Comparative transcriptomics of anal fin pigmentation patterns in cichlid fishes. *BMC Genomics* 17:712. 10.1186/s12864-016-3046-y 27600936 PMC5012078

[B77] Santos M. E. Braasch I. Boileau N. Meyer B. S. Sauteur L. Bohne A. (2014). The evolution of cichlid fish egg-spots is linked with a cis-regulatory change. *Nat. Commun.* 5:5149. 10.1038/ncomms6149 25296686 PMC4208096

[B78] Schindelin J. Arganda-Carreras I. Frise E. Kaynig V. Longair M. Pietzsch T. (2012). Fiji: an open-source platform for biological-image analysis. *Nat. Methods* 9 676–682. 10.1038/nmeth.2019 22743772 PMC3855844

[B79] Schonthaler H. B. Lampert J. M. Von Lintig J. Schwarz H. Geisler R. Neuhauss S. C. (2005). A mutation in the silver gene leads to defects in melanosome biogenesis and alterations in the visual system in the zebrafish mutant fading vision. *Dev. Biol.* 284 421–436. 10.1016/j.ydbio.2005.06.001 16024012

[B80] Seehausen O. Mayhew P. J. Van Alphen J. J. M. (1999). Evolution of colour patterns in East African cichlid fish. *J. Evol. Biol.* 12 514–534. 10.1046/j.1420-9101.1999.00055.x

[B81] Selz Y. Braasch I. Hoffmann C. Schmidt C. Schultheis C. Schartl M. (2007). Evolution of melanocortin receptors in teleost fish: the melanocortin type 1 receptor. *Gene* 401 114–122. 10.1016/j.gene.2007.07.005 17707598

[B82] Shainer I. Buchshtab A. Hawkins T. A. Wilson S. W. Cone R. D. Gothilf Y. (2017). Novel hypophysiotropic AgRP2 neurons and pineal cells revealed by BAC transgenesis in zebrafish. *Sci. Rep.* 7:44777. 10.1038/srep44777 28317906 PMC5357965

[B83] Singh A. P. Nusslein-Volhard C. (2015). Zebrafish stripes as a model for vertebrate colour pattern formation. *Curr. Biol.* 25 R81–R92. 10.1016/j.cub.2014.11.013 25602311

[B84] Singh A. P. Schach U. Nusslein-Volhard C. (2014). Proliferation, dispersal and patterned aggregation of iridophores in the skin prefigure striped colouration of zebrafish. *Nat. Cell Biol.* 16 607–614. 10.1038/ncb2955 24776884

[B85] Streelman J. T. Albertson R. C. Kocher T. D. (2003). Genome mapping of the orange blotch colour pattern in cichlid fishes. *Mol. Ecol.* 12 2465–2471. 10.1046/j.1365-294x.2003.01920.x 12919484

[B86] Theos A. C. Truschel S. T. Raposo G. Marks M. S. (2005). The Silver locus product Pmel17/gp100/Silv/ME20: controversial in name and in function. *Pigment Cell Res.* 18 322–336. 10.1111/j.1600-0749.2005.00269.x 16162173 PMC2788625

[B87] Witte F. Barel C. Witte-Maas E. L. Van Oijen M. (1976). An introduction to the taxonomy and morphology of the haplochromine Cichlidae from Lake Victoria. *Netherlands J. Zool.* 27 333–380. 10.1163/002829677x00199

[B88] Woltering J. M. Holzem M. Schneider R. F. Nanos V. Meyer A. (2018). The skeletal ontogeny of *Astatotilapia burtoni* - a direct-developing model system for the evolution and development of the teleost body plan. *BMC Dev. Biol.* 18:8. 10.1186/s12861-018-0166-4 29614958 PMC5883283

[B89] Zhang C. Song Y. Thompson D. A. Madonna M. A. Millhauser G. L. Toro S. (2010). Pineal-specific agouti protein regulates teleost background adaptation. *Proc. Natl. Acad. Sci. U.S.A.* 107 20164–20171. 10.1073/pnas.1014941107 20980662 PMC2996689

