# Supplementary material for: Developmental and Cellular Basis of Vertical Bar Color Patterns in the East African Cichlid Fish Haplochromis latifasciatus
Source: Front Cell Dev Biol. 2020 Feb 11;8:62. doi: 10.3389/fcell.2020.00062 (PMC7026194; doi:10.3389/fcell.2020.00062)
Supplement: Supplementary file 1 [file Data_Sheet_1.pdf]

Supplementary Material

Developmental and cellular basis of vertical bar color patterns in the cichlid fish *Haplochromis latifasciatus*

Running title: Vertical bar in African cichlids

Yipeng Liang[1], Jan Gerwin[1], Axel Meyer[1], Claudius F. Kratochwil[1*]

[1] Zoology and Evolutionary Biology, Department of Biology, University of Konstanz, Konstanz, Germany

*** Correspondence:**
Claudius F. Kratochwil
Claudius.Kratochwil@uni-konstanz.de

Supplementary Tables

Supplementary Table S1. Chromatophore measurement and results of the statistical tests.

Skin	Melanophore density (cells/mm²)				ANOVA		Tukey HSD *P* value						vb / ib fold change
	ib1	vb2	ib2	vb3	F-value	*P* value	ib1 *vs.* vb2	ib1 *vs.* vb3	ib2 *vs.* vb2	ib2 *vs.* vb3	vb2 *vs.* vb3	ib1 *vs.* ib2	
Individual 1	114.98 ± 41.76	362.29 ± 92.58	80.86 ± 86.86	323.73 ± 118.23	12.86	<0.001	<0.01	<0.01	<0.001	<0.01	0.902	0.929	3.50
Individual 2	131.25 ± 119.36	332.08 ± 125.54	89.51 ± 85.10	330.33 ± 86.07	7.41	<0.01	<0.05	<0.05	<0.05	<0.05	1.000	0.923	3.00
Individual 3	154.42 ± 62.51	318.21 ± 100.85	121.19 ± 94.19	333.15 ± 73.00	8.477	<0.01	<0.05	<0.05	<0.01	<0.01	0.992	0.923	2.36
**Average**	133.55 ± 19.82	337.52 ± 22.54	97.19 ± 21.23	329.07 ± 4.83	140.2	<0.001	<0.001	<0.001	<0.001	<0.001	0.942	0.154	2.89

Scale	Melanophore density (cells/mm²)				ANOVA		Tukey HSD *P* value						vb / ib fold change
	ib1	vb2	ib2	vb3	F-value	*P* value	ib1 *vs.* vb2	ib1 *vs.* vb3	ib2 *vs.* vb2	ib2 *vs.* vb3	vb2 *vs.* vb3	ib1 *vs.* ib2	
Individual 1	33.63 ± 23.80	152.95 ± 32.72	15.89 ± 16.62	157.50 ± 27.91	85.12	<0.001	<0.001	<0.001	<0.001	<0.001	0.979	0.431	6.27
Individual 2	29.79 ± 19.03	145.21 ± 42.12	77.58 ± 56.25	160.16 ± 36.51	22.22	<0.001	<0.001	<0.001	<0.01	<0.001	0.844	0.059	2.84
Individual 3	88.53 ± 60.31	163.93 ± 28.47	65.69 ± 45.14	172.52 ± 26.07	16.26	<0.001	<0.01	<0.001	<0.001	<0.001	0.967	0.636	2.18
**Average**	50.65 ± 32.86	154.03 ± 9.41	53.05 ± 32.73	163.39 ± 8.01	19.91	<0.001	<0.01	<0.01	<0.01	<0.01	0.962	0.999	3.06

Scale	Xanthophore density (cells/mm²)				ANOVA		Tukey HSD *P* value						vb / ib fold change
	ib1	vb2	ib2	vb3	F-value	*P* value	ib1 *vs.* vb2	ib1 *vs.* vb3	ib2 *vs.* vb2	ib2 *vs.* vb3	vb2 *vs.* vb3	ib1 *vs.* ib2	
Individual 1	181.35 ± 49.39	176.87 ± 39.67	142.62 ± 42.65	162.23 ± 36.49	1.702	0.184	0.995	0.744	0.285	0.730	0.866	0.190	1.05
Individual 2	121.29 ± 44.40	153.90 ± 22.90	156.08 ± 55.80	127.40 ± 30.71	1.954	0.138	0.289	0.987	0.999	0.400	0.469	0.237	1.01
Individual 3	182.85 ± 60.75	149.71 ± 35.88	157.20 ± 50.45	158.37 ± 33.85	0.907	0.448	0.411	0.658	0.983	1.000	0.975	0.624	0.91
**Average**	161.83 ± 35.12	160.16 ± 14.62	151.97 ± 8.11	149.33 ± 19.09	0.238	0.867	1.000	0.892	0.965	0.999	0.925	0.942	0.99

**Skin**	**Melanophore dispersed diameter (µm)**				**ANOVA**		**Tukey HSD *P* value**						**vb / ib fold change**
	**ib1**	**vb2**	**ib2**	**vb3**	**F-value**	***P* value**	**ib1 *vs.* vb2**	**ib1 *vs.* vb3**	**ib2 *vs.* vb2**	**ib2 *vs.* vb3**	**vb2 *vs.* vb3**	**ib1 *vs.* ib2**	
Individual 1	48.22 ± 36.23	98.94 ± 30.746	26.74 ± 27.46	99.72 ± 22.40	148.1	<0.001	<0.001	<0.001	<0.001	<0.001	0.998	<0.001	2.65
Individual 2	22.96 ± 13.31	68.87 ± 21.27	24.27 ± 23.40	70.04 ± 21.36	171.7	<0.001	<0.001	<0.001	<0.001	<0.001	0.977	0.968	2.94
Individual 3	29.68 ± 21.34	66.80 ± 31.60	48.63 ± 28.82	88.20 ± 30.68	77.66	<0.001	<0.001	<0.001	<0.001	<0.001	<0.001	<0.001	1.98
**Average**	36.20 ± 13.08	78.20 ± 17.99	33.21 ± 13.41	85.99 ± 14.96	10.69	<0.01	<0.05	<0.05	<0.05	<0.05	0.917	1.000	2.37

**Scale**	**Melanophore dispersed diameter (µm)**				**ANOVA**		**Tukey HSD *P* value**						**vb / ib fold change**
	**ib1**	**vb2**	**ib2**	**vb3**	**F-value**	***P* value**	**ib1 *vs.* vb2**	**ib1 *vs.* vb3**	**ib2 *vs.* vb2**	**ib2 *vs.* vb3**	**vb2 *vs.* vb3**	**ib1 *vs.* ib2**	
Individual 1	13.95 ± 8.78	56.47 ± 24.560	12.47 ± 5.81	65.28 ± 30.41	111.4	<0.001	<0.001	<0.001	<0.001	<0.001	<0.05	0.988	4.61
Individual 2	12.36 ± 7.78	41.09 ± 25.37	9.79 ± 6.56	39.62 ± 20.41	88.05	<0.001	<0.001	<0.001	<0.001	<0.001	0.934	0.764	3.64
Individual 3	39.03 ± 21.23	75.28 ± 21.06	37.99 ± 20.42	71.52 ± 20.14	91.67	<0.001	<0.001	<0.001	<0.001	<0.001	0.574	0.986	1.91
**Average**	21.78 ± 14.96	57.61 ± 17.12	20.08 ± 15.56	58.81 ± 16.91	5.327	<0.05	0.099	0.087	0.083	0.073	1.000	0.999	2.78

**Skin**	**Xanthophore dispersed diameter (µm)**				**ANOVA**		**Tukey HSD *P* value**						**vb / ib fold change**
	**ib1**	**vb2**	**ib2**	**vb3**	**F-value**	***P* value**	**ib1 *vs.* vb2**	**ib1 *vs.* vb3**	**ib2 *vs.* vb2**	**ib2 *vs.* vb3**	**vb2 *vs.* vb3**	**ib1 *vs.* ib2**	
Individual 1	21.15 ± 9.15	23.75 ± 9.48	28.93 ± 9.86	21.39 ± 7.75	12.61	<0.001	0.271	0.998	<0.01	<0.001	0.356	<0.001	0.90
Individual 2	21.91 ± 10.38	19.70 ± 12.52	30.59 ± 12.22	25.54 ± 12.43	12.73	<0.001	0.644	0.220	<0.001	<0.05	<0.05	<0.001	0.86
Individual 3	19.59 ± 9.78	13.55 ± 9.96	27.56 ± 11.57	21.91 ± 10.91	24.04	<0.001	<0.01	0.507	<0.001	<0.01	<0.001	<0.001	0.75
**Average**	20.88 ± 1.185	19.00 ± 5.14	29.03 ± 1.51	22.95 ± 2.26	6.455	<0.05	0.862	0.829	<0.05	0.132	0.416	<0.05	0.84

**Scale**	**Xanthophore dispersed diameter (µm)**				**ANOVA**		**Tukey HSD**						**vb / ib fold change**
	**ib1**	**vb2**	**ib2**	**vb3**	**F-value**	***P* value**	**ib1 *vs.* vb2**	**ib1 *vs.* vb3**	**ib2 *vs.* vb2**	**ib2 *vs.* vb3**	**vb2 *vs.* vb3**	**ib1 *vs.* ib2**	
Individual 1	49.59 ± 15.22	34.51 ± 12.13	34.74 ± 13.98	43.86 ± 13.82	28.38	<0.001	<0.001	<0.05	0.999	<0.001	<0.001	<0.001	0.93
Individual 2	42.52 ± 13.62	39.67 ± 11.23	50.16 ± 18.46	40.48 ± 13.13	11.12	<0.001	0.498	0.747	<0.001	<0.001	0.978	<0.01	0.86
Individual 3	50.47 ± 13.75	47.24 ± 12.88	60.71 ± 16.84	56.14 ± 13.24	17.57	<0.001	0.379	<0.05	<0.001	0.108	<0.001	<0.001	0.93
**Average**	47.53 ± 4.36	40.47 ± 6.40	48.54 ± 13.06	46.83 ± 8.24	0.535	0.671	0.754	1.000	0.675	0.995	0.805	0.999	0.91

Skin	Melanophore coverage (%)				ANOVA		Tukey HSD *P* value						vb / ib fold change
	ib1	vb2	ib2	vb3	F-value	*P* value	ib1 *vs.* vb2	ib1 *vs.* vb3	ib2 *vs.* vb2	ib2 *vs.* vb3	vb2 *vs.* vb3	ib1 *vs.* ib2	
Individual 1	7.24 ± 3.02	83.79 ± 8.64	6.31 ± 5.81	72.91 ± 9.84	161.2	<0.001	<0.001	<0.001	<0.001	<0.001	0.128	0.997	11.56
Individual 2	7.78 ± 6.62	70.76 ± 13.09	12.68 ± 16.85	69.89 ± 15.22	33.06	<0.001	<0.001	<0.001	<0.001	<0.001	1.000	0.939	6.87
Individual 3	26.17 ± 21.25	80.09 ± 7.71	22.40 ± 15.38	78.08 ± 11.77	22.67	<0.001	<0.001	<0.001	<0.001	<0.001	0.996	0.978	3.26
**Average**	13.73 ± 10.78	78.21 ± 6.72	13.80 ± 8.10	73.63 ± 4.14	63.51	<0.001	<0.001	<0.001	<0.001	<0.001	0.887	1.000	5.52

Scale	Melanophore coverage (%)				ANOVA		Tukey HSD *P* value						vb / ib fold change
	ib1	vb2	ib2	vb3	F-value	*P* value	ib1 *vs.* vb2	ib1 *vs.* vb3	ib2 *vs.* vb2	ib2 *vs.* vb3	vb2 *vs.* vb3	ib1 *vs.* ib2	
Individual 1	0.54 ± 1.04	24.80 ± 11.40	0.22 ± 0.30	28.70 ± 7.35	47.67	<0.001	<0.001	<0.001	<0.001	<0.001	0.604	1.000	70.39
Individual 2	0.51 ± 0.38	18.14 ± 15.17	0.32 ± 0.49	12.70 ± 5.41	12.32	<0.001	<0.001	<0.01	<0.001	<0.01	0.442	1.000	37.16
Individual 3	5.11 ± 5.33	38.41 ± 13.29	5.23 ± 4.99	35.74 ± 8.70	44.56	<0.001	<0.001	<0.001	<0.001	<0.001	0.903	1.000	7.17
**Average**	2.05 ± 2.65	27.12 ± 10.33	1.92 ± 2.87	25.71 ± 11.81	9.145	<0.01	<0.05	<0.05	<0.05	<0.05	0.996	1.000	13.31

Skin	Xanthophore coverage (%)				ANOVA		Tukey HSD *P* value						vb / ib fold change
	ib1	vb2	ib2	vb3	F-value	*P* value	ib1 *vs.* vb2	ib1 *vs.* vb3	ib2 *vs.* vb2	ib2 *vs.* vb3	vb2 *vs.* vb3	ib1 *vs.* ib2	
Individual 1	56.94 ± 11.93	12.62 ± 5.89	60.29 ± 8.79	13.16 ± 5.23	39.72	<0.001	<0.001	<0.001	<0.001	<0.001	1.000	0.941	0.22
Individual 2	32.00 ± 16.13	12.22 ± 9.09	44.69 ± 7.85	11.13 ± 6.52	9.457	<0.01	0.087	0.068	<0.01	<0.01	0.998	0.366	0.30
Individual 3	51.79 ± 9.60	7.74 ± 4.65	56.79 ± 11.72	8.37 ± 3.46	43.61	<0.001	<0.001	<0.001	<0.001	<0.001	0.999	0.819	0.15
**Average**	46.91 ± 13.17	10.86 ± 2.71	53.92 ± 8.18	10.89 ± 2.40	25.07	<0.001	<0.01	<0.01	<0.001	<0.001	1.000	0.711	0.22

Scale	Xanthophore coverage (%)				ANOVA		Tukey HSD *P* value						vb / ib fold change
	ib1	vb2	ib2	vb3	F-value	*P* value	ib1 *vs.* vb2	ib1 *vs.* vb3	ib2 *vs.* vb2	ib2 *vs.* vb3	vb2 *vs.* vb3	ib1 *vs.* ib2	
Individual 1	20.92 ± 5.08	18.13 ± 6.69	12.38 ± 5.71	13.53 ± 4.48	5.166	<0.01	0.678	<0.05	0.113	0.967	0.265	<0.01	0.95
Individual 2	14.29 ± 3.66	14.86 ± 4.26	12.27 ± 4.79	12.56 ± 2.66	1.055	0.380	0.988	0.757	0.462	0.998	0.562	0.660	1.03
Individual 3	22.24 ± 9.87	14.21 ± 2.68	28.26 ± 7.18	20.83 ± 8.20	5.962	<0.01	0.094	0.974	<0.001	0.136	0.214	0.290	0.69
**Average**	19.15 ± 4.26	15.73 ± 2.10	17.64 ± 9.20	15.64 ± 4.52	0.266	0.848	0.878	0.869	0.975	0.971	1.000	0.987	0.85

Supplementary Table S2. Relative expression levels of target genes and results of the statistical tests.

Gene	Expression level				ANOVA		Tukey HSD *P* values						vb / ib fold change
	ib1 (n=5)	vb2 (n=5)	ib2 (n=5)	vb3 (n=5)	F-value	*P* value	ib1 *vs.* vb2	ib1 *vs.* vb3	ib2 *vs.* vb2	ib2 *vs.* vb3	vb2 *vs.* vb3	ib1 *vs.* ib2	
sox10	1.11 ± 0.10	1.43 ± 0.32	1.18 ± 0.13	1.51 ± 0.30	3.371	<0.05	0.193	0.067	0.371	0.148	0.931	0.971	1.28
mitfa	1.51 ± 0.30	2.16 ± 0.60	1.52 ± 0.61	1.95 ± 0.58	1.787	0.19	0.268	0.573	0.283	0.595	0.933	1.000	1.36
csf1ra	1.58 ± 0.39	1.53 ± 0.38	1.24 ± 0.19	1.75 ± 0.47	1.611	0.226	0.997	0.878	0.628	0.176	0.779	0.506	1.16
ltk	2.64 ± 1.02	2.45 ± 1.03	2.01 ± 0.70	2.85 ± 0.63	0.862	0.481	0.985	0.979	0.853	0.437	0.879	0.664	1.14
pmel	2.70 ± 0.49	3.60 ± 1.37	1.75 ± 0.62	3.61 ± 1.59	3.102	0.056	0.599	0.588	0.082	0.079	1.000	0.554	1.62
slc24a5	2.05 ± 1.08	7.44 ± 3.67	2.96 ± 1.08	13.21 ± 6.88	8.267	<0.01	0.181	<0.01	0.317	<0.01	0.140	0.983	4.12
tyr	2.06 ± 0.46	10.79 ± 4.81	1.63 ± 0.63	12.49 ± 5.10	13.05	<0.001	<0.001	<0.01	<0.01	<0.001	0.872	0.997	6.31
tyrp1a	1.99 ± 0.31	11.51 ± 4.88	1.74 ± 0.63	12.35 ± 4.14	16.34	<0.001	<0.01	<0.001	<0.01	<0.001	0.975	0.999	6.40
asip1	6.55 ± 2.73	2.84 ± 0.92	3.66 ± 0.34	2.20 ± 0.86	8.052	<0.01	<0.01	<0.01	0.822	0.907	0.907	<0.05	0.49
agrp2	1.42 ± 0.61	2.15 ± 1.08	3.63 ± 1.38	4.44 ± 2.72	3.481	<0.05	0.896	<0.05	0.506	0.860	0.164	0.189	1.30
mc1r	2.72 ± 0.96	2.33 ± 1.09	3.03 ± 1.14	3.01 ± 1.45	0.388	0.764	0.952	0.978	0.785	1.000	0.795	0.975	0.93
mc5r	1.64 ± 0.91	2.39 ± 0.81	1.85 ± 0.67	1.57 ± 0.38	1.341	0.296	0.376	0.999	0.641	0.926	0.307	0.965	1.13

Supplementary Table S3. Melanin synthesis genes expression levels (divide by expression of *mitfa*) and results of the statistical tests.

Gene	Expression level				ANOVA		Tukey HSD *P* values						vb / ib fold change
	ib1 (n=5)	vb2 (n=5)	ib2 (n=5)	vb3 (n=5)	F-value	*P* value	ib1 *vs.* vb2	ib1 *vs.* vb3	ib2 *vs.* vb2	ib2 *vs.* vb3	vb2 *vs.* vb3	ib1 *vs.* ib2	
pmel	1.84 ± 0.47	1.66 ± 0.27	1.29 ± 0.53	1.84 ± 0.63	1.397	0.28	0.933	1.000	0.650	0.319	0.930	0.324	1.12
slc24a5	1.36 ± 0.68	3.37 ± 1.22	2.04 ± 0.66	6.66 ± 2.85	10.55	<0.001	0.245	<0.001	0.580	<0.01	<0.05	0.910	2.95
tyr	1.39 ± 0.35	4.89 ± 1.33	1.10 ± 0.28	6.36 ± 1.50	31.9	<0.001	<0.001	<0.001	<0.001	<0.001	0.148	0.970	4.52
tyrp1a	1.34 ± 0.22	5.24 ± 1.40	1.21 ± 0.45	6.40 ± 1.31	36.22	<0.001	<0.001	<0.001	<0.001	<0.001	0.285	0.997	4.56

Supplementary Table S4. Melanophore related genes expression levels (standardized by melanophore density)

Gene	Gene expression (Skin)				vb / ib fold change
	ib1	vb2	ib2	vb3	
mitfa	0.0113	0.0064	0.0156	0.0059	0.46
pmel	0.0202	0.0107	0.0180	0.0110	0.57
slc24a5	0.0154	0.0220	0.0305	0.0401	1.36
tyr	0.0154	0.0320	0.0168	0.0380	2.17
tyrp1a	0.0149	0.0341	0.0179	0.0375	2.18

Gene	Gene expression (Scale)				vb / ib fold change
	ib1	vb2	ib2	vb3	
mitfa	0.0298	0.0140	0.0287	0.0119	0.44
pmel	0.0533	0.0234	0.0330	0.0221	0.53
slc24a5	0.0405	0.0483	0.0558	0.0808	1.34
tyr	0.0407	0.0701	0.0307	0.0764	2.05
tyrp1a	0.0393	0.0747	0.0328	0.0756	2.09

Gene	Gene expression (Skin + Scale)				vb / ib fold change
	ib1	vb2	ib2	vb3	
mitfa	0.0164	0.0088	0.0202	0.0079	0.46
pmel	0.0293	0.0146	0.0233	0.0147	0.56
slc24a5	0.0223	0.0303	0.0394	0.0536	1.36
tyr	0.0224	0.0439	0.0217	0.0507	2.15
tyrp1a	0.0216	0.0468	0.0232	0.0502	2.17

Supplementary Table S5. Primer sequence for qPCR.

Name	Sequence (5' – 3')	Gene
agrp2_Fwd	GCGAAGAATAGGCGGCTGTTTG	*agrp2*
agrp2_Rev	CGACGCGCCGGAGTTACGAG	*agrp2*
asip1_Fwd	GAAGAGCAAGAAACCAAAGAAACA	*asip1*
asip1_Rev	ACTGGCAGAAAGCACAATAATCAC	*asip1*
csf1ra_F	GCAATGCACGTCTGCCAGTG	*csf1ra*
csf1ra_R	ACGTCACTCTGGACGGTGT	*csf1ra*
gapdh_Fwd	CACACAAGCCCAACCCATAGTCAT	*gapdh*
gapdh_Rev	AAACACACTGCTGCTGCCTACATA	*gapdh*
ltk_F	GCCACAGCAACCACAGAGTAC	*ltk*
ltk_R	GAAGTGTCCATCAGACCTTCCCTC	*ltk*
mc1r_Fwd	TGCTGGGGCCCTTTCTTTCTACAC	*mc1r*
mc1r_Rev	AAGCGGGTCGATGAGGGAGTTACA	*mc1r*
mc5r_Fwd	ATCCTGGGTATCATCTCACTGC	*mc5r*
mc5r_Rev	CATGTCTGCTACTGCCAAACTG	*mc5r*
mitfa_Fwd	CGACGATGTTCTTGGATTGATGGA	*mitfa*
mitfa_Rev	CGAGGCCTGGTAGCTGGAGACTT	*mitfa*
pmel_F	CAGTTCTCCATCACTGATCAAATCCC	*pmel*
pmel_R	TCGCCCTGTTCTGGATGAAGC	*pmel*
slc24a5_F	CACCTGAACTAGTCACAGCCTTCC	*slc24a5*
slc24a5_R	TGAGACGCCCAGCCATAGA	*slc24a5*
sox10_Fwd	AGCGGCGAGGAGGAAACTTG	*sox10*
sox10_Rev	GAATGGCCTCTTGTCGGTCTCA	*sox10*
tyr_Fwd	TCCCTTAATCCCAACCTCATCAGC	*tyr*
tyr_Rev	ACCCTCCCCCGTGGCATTACATA	*tyr*
tyrp1a_Fwd	GCGGGGCAGGCCAGTTTTG	*tyrp1a*
tyrp1a_Rev	GGCAGGGCGAAGGAAGGGTTTT	*tyrp1a*
β-actin_Fwd	TGACATGGAGAAGATCTGGC	*β-actin*
β-actin_Rev	TGGCAGGAGTGTTGAAGGT	*β-actin*

Supplementary Figures

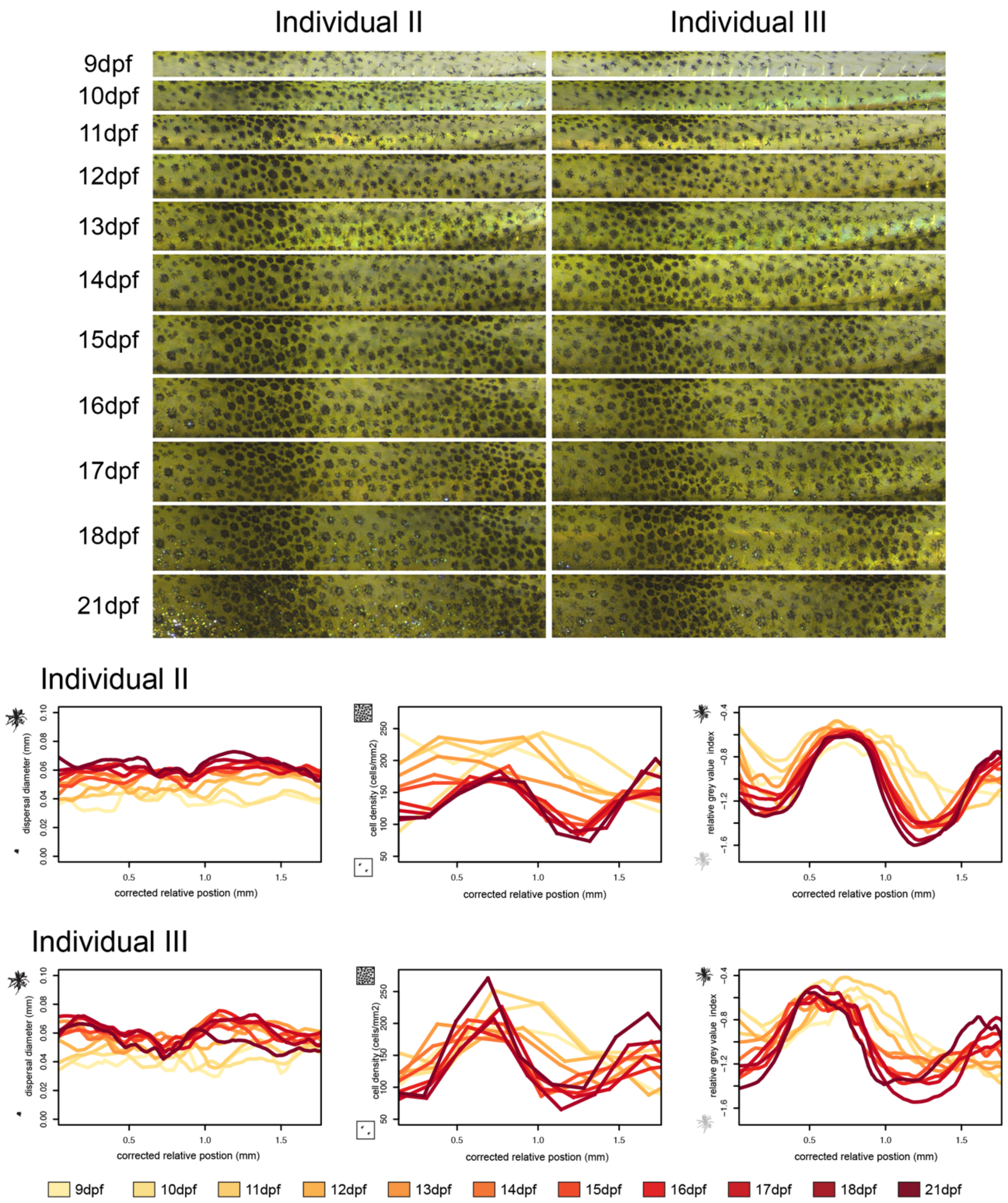

Supplementary Figure S1. Documentation of vertical bar formation (top) and quantifications (bottom) in two additional individuals.

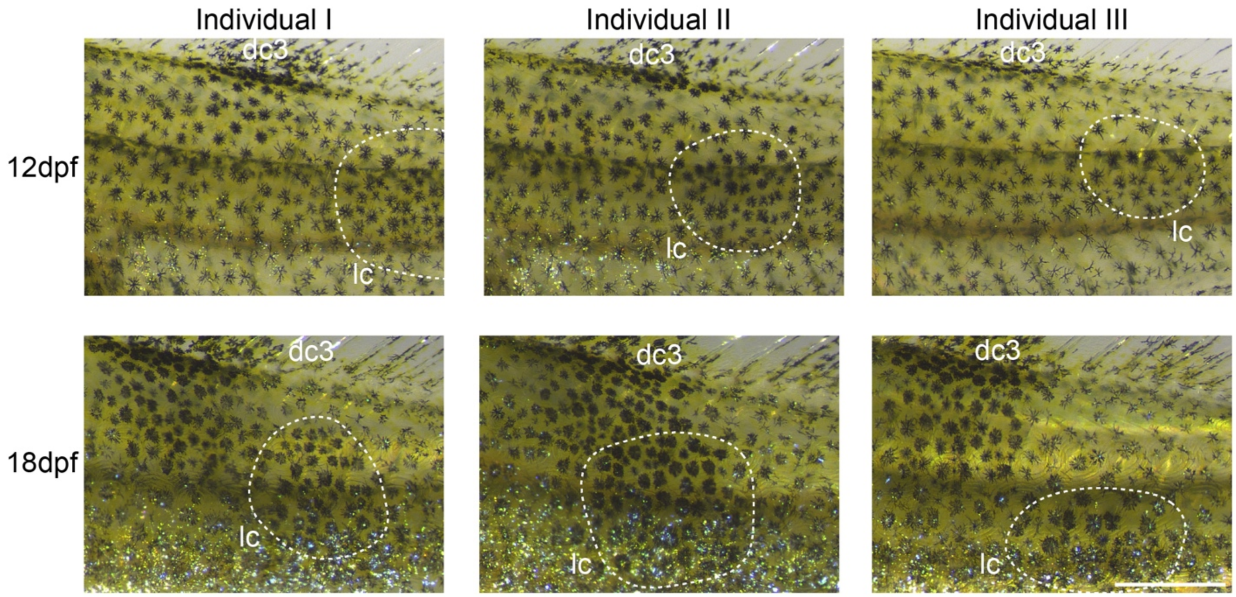

Supplementary Figure S2. Detailed photographs of lateral cluster. A new melanophore cluster (lateral cluster, lc, dotted outline in all panels) as a distinct entity form around 12dpf in all assessed individuals. The lc merges with dc3 and develops into the third vertical bar (vb3).

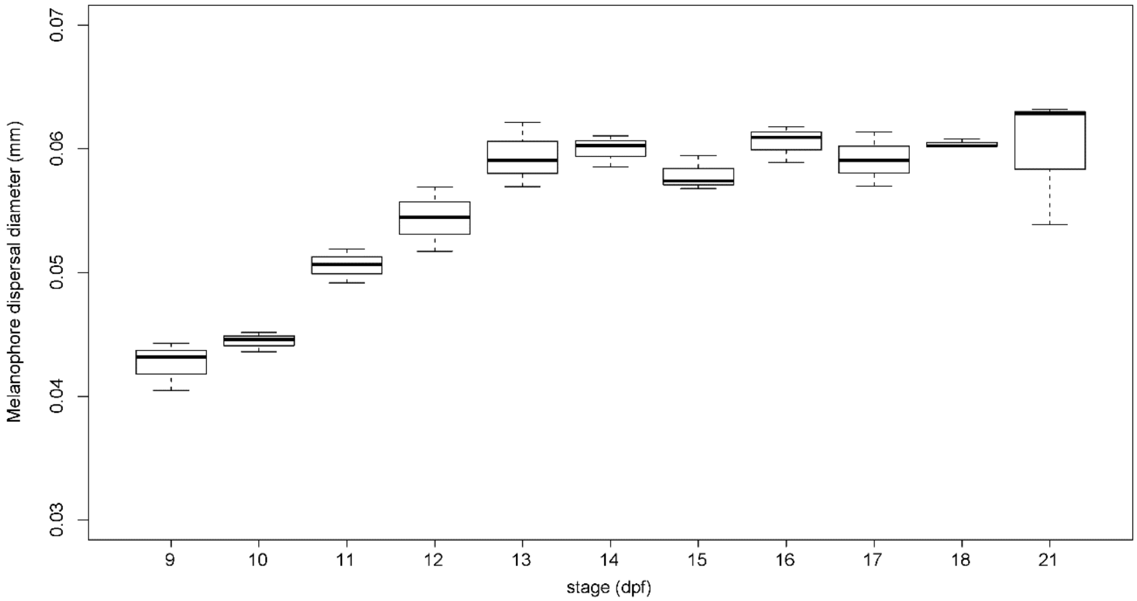

Supplementary Figure S3. Changes in melanophore dispersal diameter over time suggesting an increase in cell size at early stages (between 9 and 13 dpf).

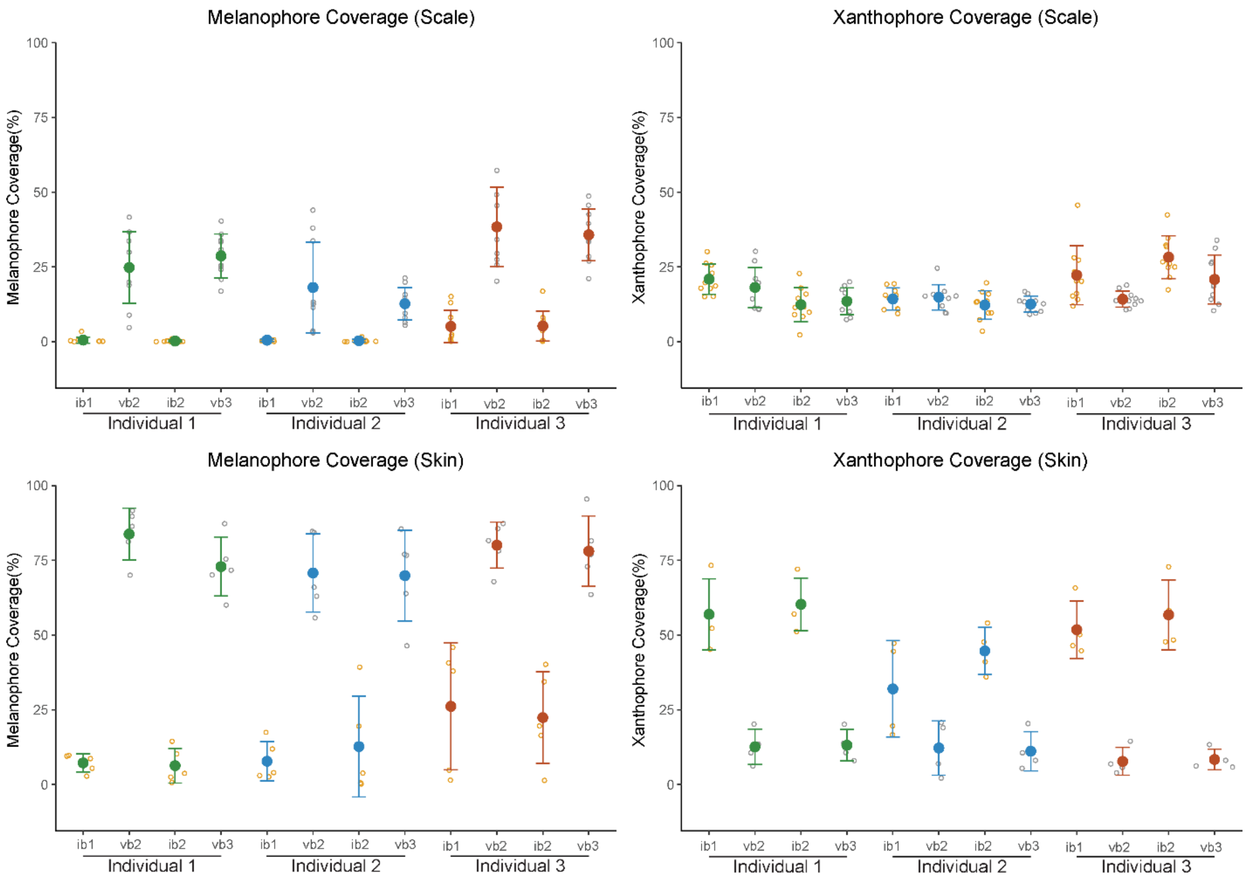

Supplementary Figure S4. Pigment coverage of the three individuals. Each hollow point represents measurement from one region. Solid points represent the mean value of these. Error bars indicate means ± SD.

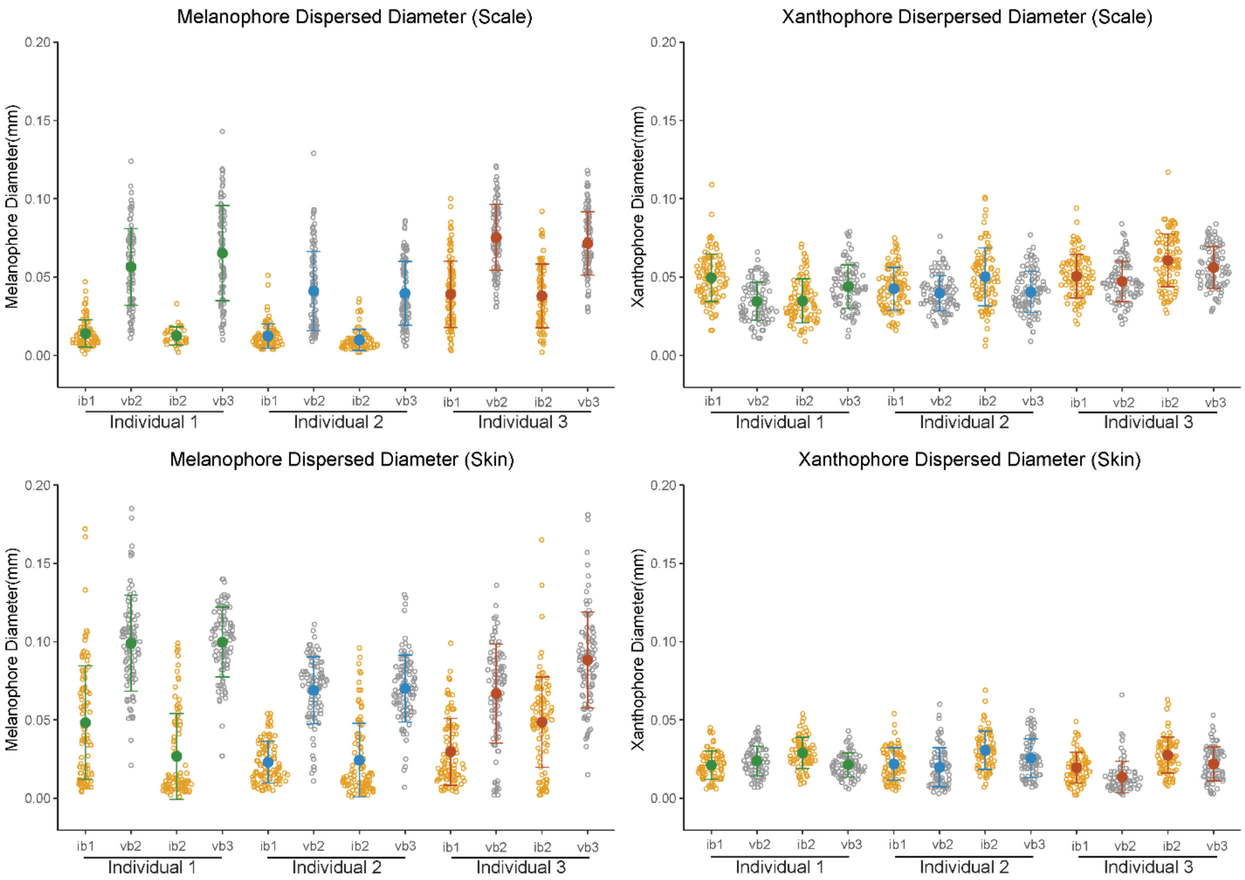

Supplementary Figure S5. Chromatophore dispersal diameter in the three individuals. Each hollow point represents one measured cell. Solid points represent the mean value of these. Error bars indicate means ± SD.

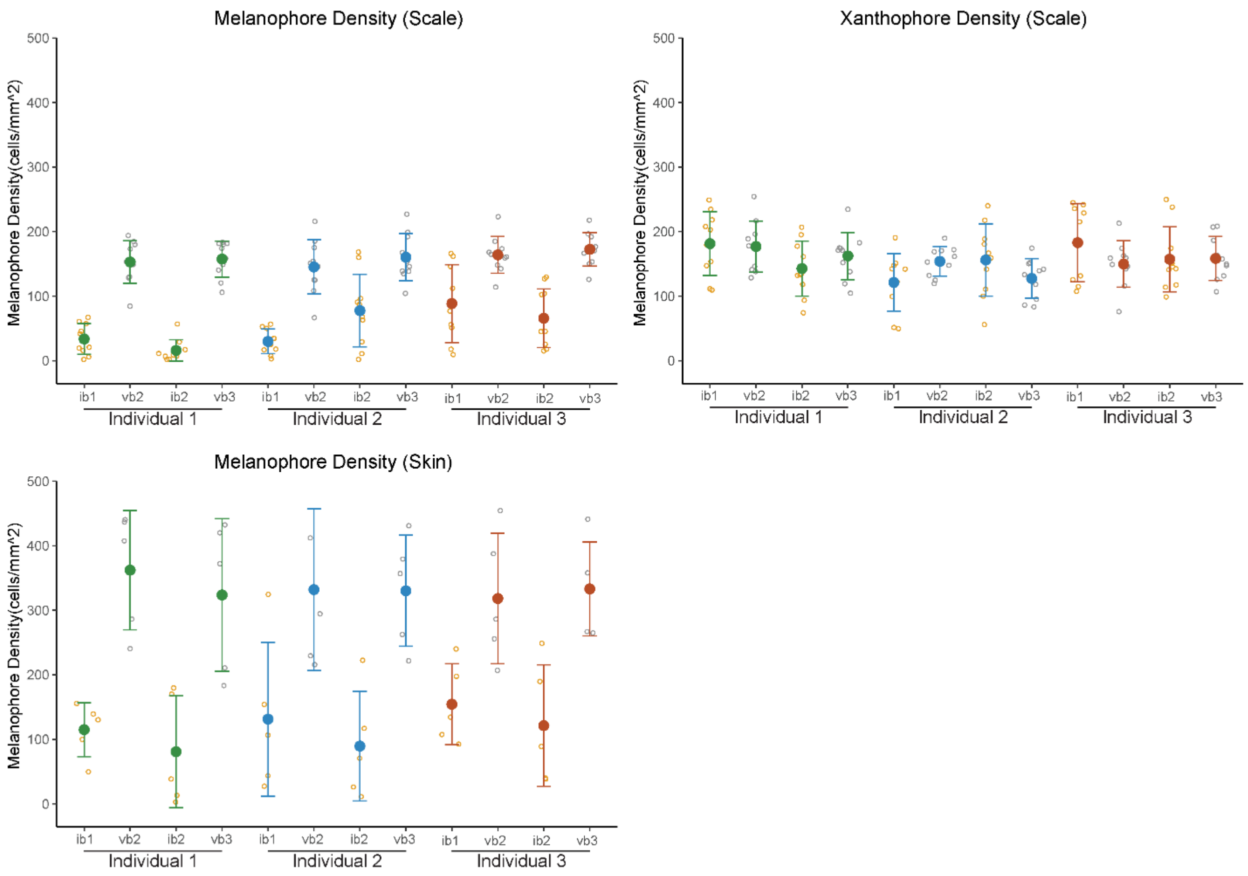

Supplementary Figure S6. Chromatophore density in the three individuals. Each hollow point represents measurement from one region. Solid points represent the mean value of these. Error bars indicate means ± SD.

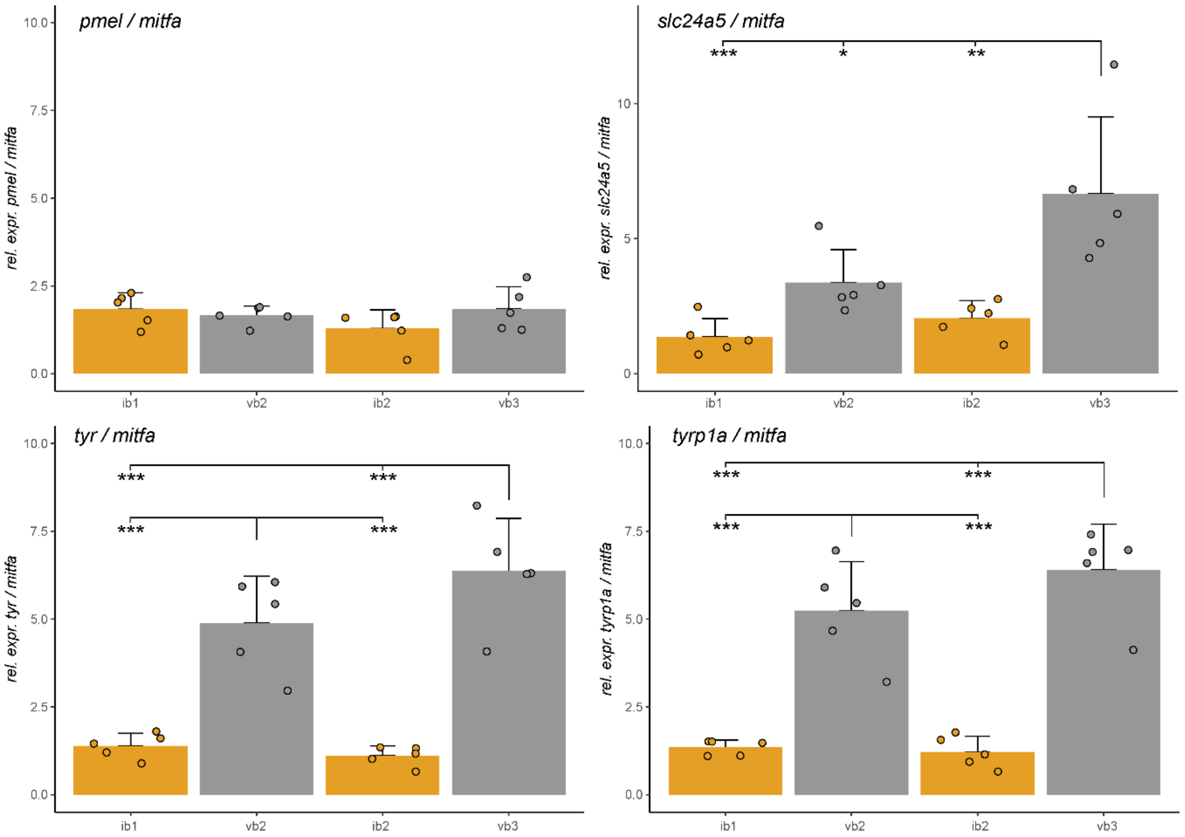

Supplementary Figure S7. Relative expression of melanophore genes corrected by relative expression of *mitf*. Differences were tested by ANOVA followed by Tukey–Kramer post-hoc test, n = 5 (individual dots). Error bars indicate means + SD. Abbreviations: ***, P<0.001; **, P<0.01; *, P<0.05.
